# Applications of Machine Learning in the Research of Heavy Metal(loid)s-Related Risk: A Scoping Review of Methodology

**DOI:** 10.3390/toxics14080683

**Published:** 2026-08-03

**Authors:** Zhuang Liu, Yonghai Gan, Chengcheng Ding, Zheng Wang, Jiabao Yan, Rujiao Tan, Yang Li, Jun Luo, Yibin Cui

**Affiliations:** 1 Nanjing Institute of Environmental Sciences, Ministry of Ecology and Environment, Nanjing 210042, China; liuzhuang@nies.org (Z.L.); ganyonghai@nies.org (Y.G.); dcc@nies.org (C.D.); wangzheng1555@126.com (Z.W.); jiabao.yan@foxmail.com (J.Y.); 2 Tianjin Municipal Engineering Design & Research Institute, Tianjin 300392, China; tanrujiao@163.com; 3 Unmanned System Research Institute, Northwestern Polytechnical University, Xi’an 710129, China; liyangusri@nwpu.edu.cn

**Keywords:** machine learning, heavy metal, human health risk, ecological risk

## Abstract

The rapid expansion of machine learning applications in heavy metal risk research has generated a large but fragmented body of literature, necessitating a systematic summary of various methodologies. This survey examines 182 research articles from Web of Science, Scopus, and IEEE Xplore over the past 10 years, providing a comprehensive analysis of the application of prevalent machine learning algorithms across research areas related to heavy metal risks, covering more than 30 algorithms and 9 research areas. The results show that regional risk assessment, risk source analysis, risk driver analysis, and research on the pathogenicity of HMs are the four most frequently applied areas of machine learning, accounting for 80.06% of all applications. Classical machine learning, especially a series of tree-based algorithms, dominates across all applications, accounting for 69.93% and 42.74%, respectively. In addition, some auxiliary algorithms, particularly those for feature analysis, are often used in conjunction with machine learning, primarily to interpret model predictions and analyze risk sources or drivers. Three principal methodological challenges emerge from our review: (1) poor model generalizability across different environmental conditions; (2) insufficient reliability of predictions; and (3) difficulty in obtaining high-quality training data. Some current literature is also constrained by small sample sizes, regional bias, limited field validation, and insufficient integration of multi-omics data with machine learning pipelines. To address these gaps, we advocate for the routine adoption of explainable AI techniques with rigorous stability checks, the development of publicly available, field-validated benchmark datasets, and greater integration of mechanistic knowledge with data-driven models.

## 1. Introduction

Heavy metals generally refer to metals with high density, atomic number, or atomic weight [1,2], and some metalloids such as As are also included due to their pronounced toxicity [3,4]; numerous studies refer to them as heavy metal(loid)s (HMs) [5,6]. HMs are recognized as significant environmental pollutants even at low concentrations due to their toxicity, persistence, and bioaccumulation [7,8], affecting ecological systems and human health through water, soil, and air [9,10,11], and are one of the main sources of ecological and human health risks [12,13,14,15]. HMs can pose a threat to humans and other organisms [7,16], and they not only directly damage organisms and ecosystems [17,18,19] but also cause chronic or acute damage to human organs and pose potential carcinogenic and teratogenic risks [20]. For example, Pb can cause serious damage to basic cellular processes, kidneys, brain, and liver [21]; Cd can lead to damage to the kidneys, bones, lungs, and liver [22]; and Hg exposure may lead to central nervous system defects, cardiomyopathy, arrhythmias, kidney damage, and respiratory failure [23].

Research on HM-related risks often involves highly complex exposure scenarios. Many geographical and environmental factors (such as temperature, pH, topography, hydrology, and soil properties) affect the environmental behavior and fate of HMs [24,25,26]. The intake and pathogenicity of HMs are closely related to the physiological and sociodemographic features of the population [27,28]. These features often exhibit complex nonlinear relationships among themselves and with the risk level. Therefore, risk assessment and analysis need to comprehensively consider the characteristics of the environment, demographics, and socioeconomic factors, and address the complex nonlinear relationships among variables [29,30]. Traditional risk analysis methods (such as linear extrapolation of dose–response effects) mainly rely on predefined linear models, fixed parameters, and numerous simplifying assumptions (such as ignoring multi-factor interactions and assuming that exposure parameters follow a standard normal distribution) [31]. These faults make it difficult to describe how risk sources and receptors interact accurately, how different HMs may act together or in opposition, and how receptors respond differently. As a result, risk assessment and analysis become less reliable [32].

Machine learning has come to play an important role in the study of HM-related risks due to its ability to automatically learn high-dimensional nonlinear relationships, process multi-source heterogeneous data, and identify potential interaction terms and key driving factors [33,34,35]. It has shown significant advantages across multiple areas: in risk assessment and prediction, it overcomes limitations of traditional linear models to improve accuracy and efficiency [36,37]; in risk source identification, it separates contribution ratios of different sources from mixed signals without prior spectral assumptions [38,39]; in risk driver analysis, it uses feature-importance rankings to automatically distinguish natural and anthropogenic factors and reveal hidden nonlinear mechanisms [4,40]; in studying HM toxicity and stress effects, it captures subtle concentration-response patterns and predicts organism responses [41,42,43,44]; in biomarker discovery, its dimensionality reduction and pattern-recognition capabilities screen massive omics data to identify sensitive and robust biomarker combinations [45,46]; and in spatial risk pattern analysis, it integrates methods like Geographically Weighted Regression and Kriging to accurately characterize spatial patterns [47,48]. Overall, by flexibly learning the inherent structure of data rather than imposing pre-defined formulas, machine learning enables a leap from local linear thinking to global nonlinear system cognition in HM-related risk research.

Machine learning has been extensively applied in HM research, and several reviews have synthesized these efforts from various angles [49,50,51]. However, the existing reviews predominantly address pollution control and remediation strategies, with relatively limited attention to the full chain of HM-related risk research—from exposure concentration prediction to toxicity and health effect assessment. Moreover, these reviews tend to summarize applications descriptively, without critically evaluating the methodological performance of different machine learning algorithms across diverse risk prediction tasks, nor do they systematically examine the fundamental challenges. Consequently, a critical gap remains: while the volume of machine learning applications in HM risk research is rapidly expanding, the field lacks a clear, evidence-based overview of what has been achieved, which approaches perform best under what conditions, and what methodological and data-related obstacles must be overcome to advance the field. To fill this gap, this scoping review provides a systematic synthesis of research cases on machine learning applications in HM risk research. Specifically, we attempt to answer three research questions: (1) What are the commonly used machine learning-related algorithms in heavy metal risk research, and what are their characteristics and applicable scenarios? (2) In which heavy metal-related risk research areas is machine learning mainly applied, and how is it applied? (3) What challenges does current research face? What are the future research directions?

## 2. Review Methodology

### 2.1. Overall Review Strategy

This scoping review follows the Preferred Reporting Items for Systematic Reviews and Meta-Analyses Extension for Scoping Reviews (PRISMA-ScR) guidelines [52,53]. A comprehensive literature search was conducted on 6 March 2026 across the Web of Science, Scopus, and IEEE Xplore, using search strings that combined terms related to heavy metals, risk, and machine learning (detailed in Section 2.3 and Table 1). All retrieved records were imported into Zotero, a reference management system. After removing duplicates through automated deduplication, two independent reviewers manually screened the titles, abstracts, and full texts against predefined eligibility criteria to determine the final set of included studies. Any disagreements during screening were resolved through discussion or consultation with a third reviewer.

### 2.2. Eligibility Criteria

Studies were included if they met all of the following criteria: (1) employed machine learning, deep learning, or artificial intelligence algorithms for data analysis or prediction; (2) addressed risks associated with heavy metals, encompassing both ecological risks and human health risks; (3) published between 2016 and 2026, a period marked by the rapid proliferation of machine learning applications in environmental research; and (4) reviews, editorials, conference abstracts, notes, and book chapters were excluded to maintain consistency and quality of synthesized evidence.

### 2.3. Search Strategy

The search strategy was designed to maximize the retrieval of relevant studies on machine learning applications in HM-related risk research. Core search terms comprised three conceptual groups: (1) heavy metal-related terms: “heavy metal” and the eight most common metal(loid)s—”cadmium”, “lead”, “arsenic”, “chromium”, “mercury”, “nickel”, “copper”, and “zinc”; (2) risk-related terms: “risk”, “hazard”, “toxicity”, “exposure”, “safety”, and “health”; (3) machine learning-related terms: “machine learning”, “deep learning”, “artificial intelligence”, “neural network”. These terms were combined using Boolean operators (AND/OR) with field-specific search syntax adapted to each database. The detailed search strings are provided in Table 1. To ensure comprehensiveness, reference lists of included reviews were also manually cross-checked for additional relevant primary studies not captured by the database search.

### 2.4. Selection of Sources of Evidence

**Figure 1:**
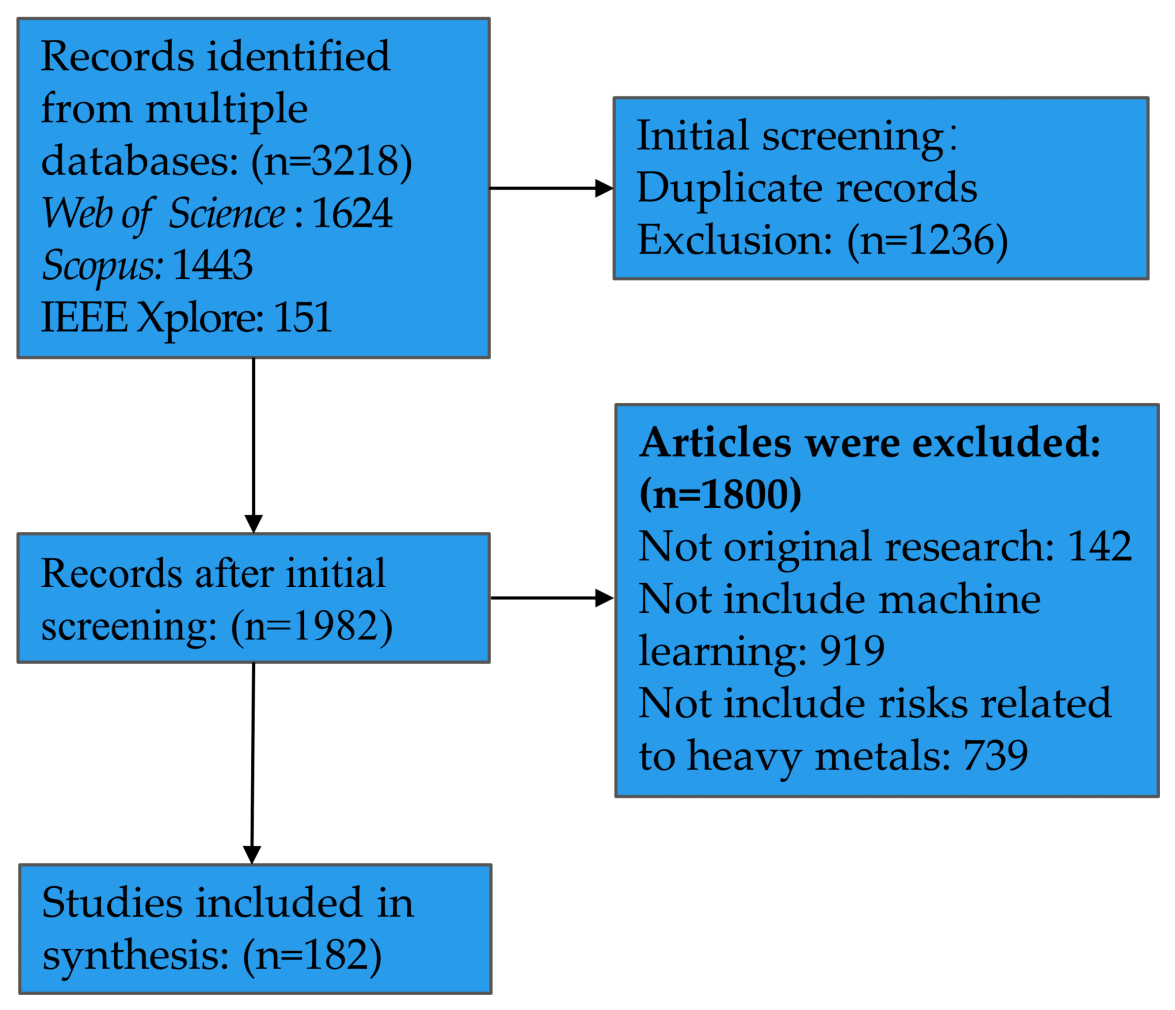
Flow diagram of the screening and selection process.

All records retrieved from databases based on search strings were imported into the Zotero. The system automatically identified and removed duplicate records, obtaining initial screening results. The initial screening results still included many records that were inconsistent with the focus of this review. Therefore, further manual screening was required. In this process, two reviewers independently assessed the final eligibility for inclusion of each record. Disagreements were resolved through discussion; if no consensus could be reached, a third reviewer will participate in the decision. Through three rounds of screening—title, abstract, and full text—182 closely related papers were ultimately obtained. The literature screening process is shown in Figure 1.

### 2.5. Data Extraction

Data extraction was performed using pre-designed structured forms to ensure consistency and accuracy across studies. The extraction forms included fields such as authors and publication year, types of risks, research area, and algorithms used in the study. Because each paper often involves more than one research area and employs multiple algorithms, one-hot codes are used for labeling. If a paper involves a specific research area or uses a specific algorithm, the corresponding column is labeled 1; otherwise, it is labeled 0.

### 2.6. Data Synthesis

We grouped the machine learning algorithms by technical characteristics and categorized them into early machine learning, classical machine learning, and deep learning, as well as the algorithms for feature analysis and risk probability calculation. The data obtained from the survey were statistically analyzed and summarized in Excel, and various statistical charts were created in Origin, including bar, sunburst, and pie charts. The project team assigned a dedicated person to implement the data analysis and visualization.

## 3. Introduction to Machine Learning-Related Algorithms Commonly Used in Heavy Metal Risk Research

Machine learning is a technique that improves computer systems through experience (i.e., data). It automatically learns inherent patterns from training data and then uses them to classify, predict, or make other decisions about unseen data [54,55]. Machine learning, deep learning, and artificial intelligence are sometimes used interchangeably in some literature. Artificial intelligence is the broadest concept, referring to the entire technological field that enables machines to simulate human intelligent behavior. Machine learning is a core branch of artificial intelligence, with the basic idea of allowing computers to automatically learn patterns and rules from data rather than relying on explicitly programmed rules. Deep learning, on the other hand, is a subset that specifically refers to methods that use multi-layered neural networks to automatically extract hierarchical features from large amounts of data. Machine learning has generally gone through the development stages of early machine learning, classical machine learning, deep learning, and reinforcement learning.

### 3.1. Taxonomy

**Figure 2:**
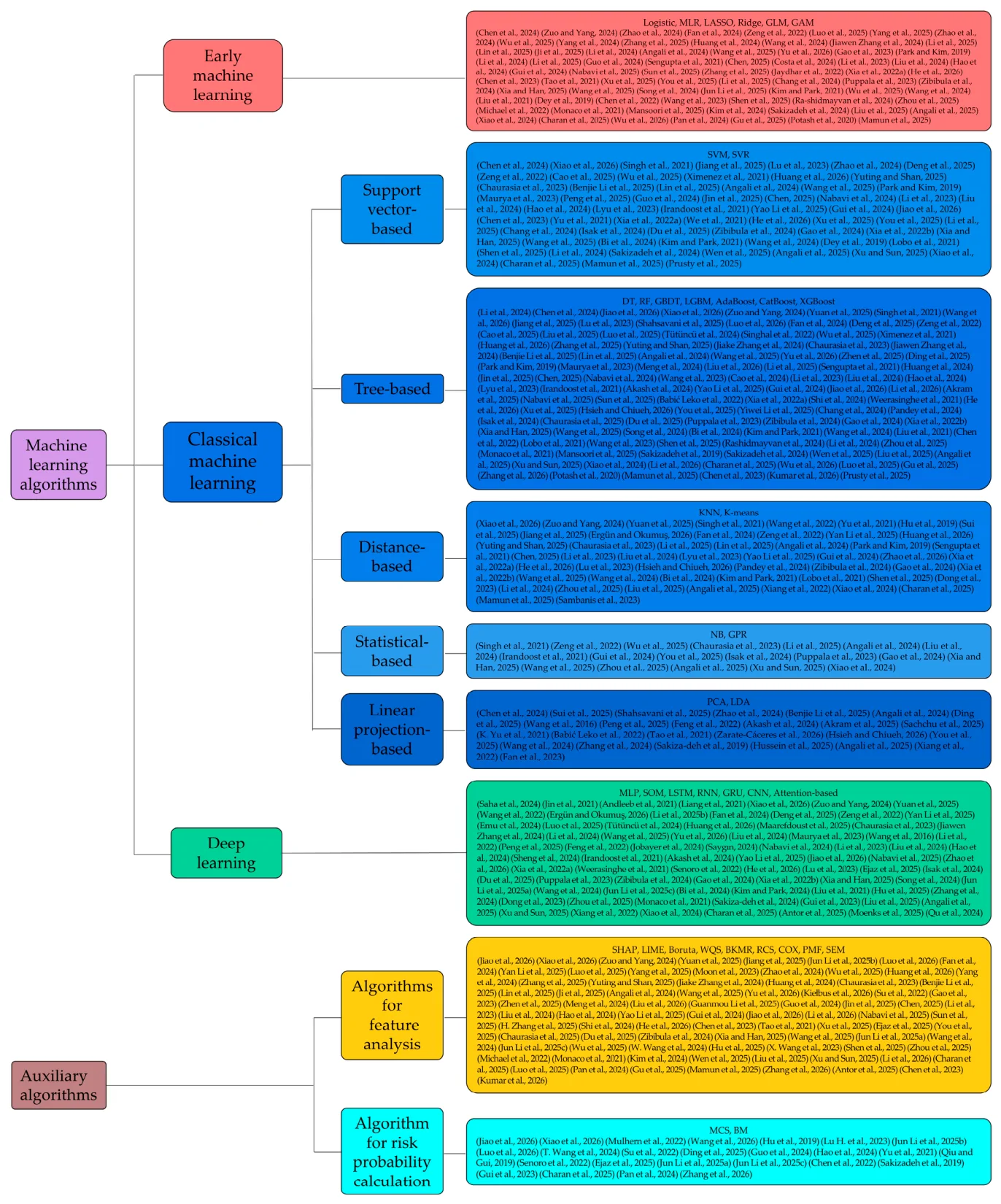
Machine learning-related algorithms commonly used in heavy metal risks research [4,6,24,25,26,27,28,29,30,32,33,34,35,36,37,38,39,40,41,42,43,44,45,46,47,48,49,56,57,58,59,60,61,62,63,64,65,66,67,68,69,70,71,72,73,74,75,76,77,78,79,80,81,82,83,84,85,86,87,88,89,90,91,92,93,94,95,96,97,98,99,100,101,102,103,104,105,106,107,108,109,110,111,112,113,114,115,116,117,118,119,120,121,122,123,124,125,126,127,128,129,130,131,132,133,134,135,136,137,138,139,140,141,142,143,144,145,146,147,148,149,150,151,152,153,154,155,156,157,158,159,160,161,162,163,164,165,166,167,168,169,170,171,172,173,174,175,176,177,178,179,180,181,182,183,184,185,186,187,188,189,190,191,192,193,194,195,196,197,198,199,200,201,202,203,204,205,206,207,208,209,210].

Figure 2 presents the taxonomy of machine learning-related algorithms commonly used in HM risk research. According to the literature survey, more than 20 commonly used machine learning algorithms are currently used in HM risk research, which can be broadly divided into three categories: early machine learning, classical machine learning, and deep learning. Among these, early machine learning algorithms include Logistic Regression (Logistic), Multiple Linear Regression (MLR), LASSO Regression (LASSO), Ridge Regression, Generalized Linear Model (GLM), and Generalized Additive Models (GAM).

Deep learning algorithms mainly include Multilayer Perceptron (MLP), Self-Organizing Map (SOM), Long Short-Term Memory (LSTM), Recurrent Neural Networks (RNNs), Gated Recurrent Units (GRUs), Convolutional Neural Networks (CNN), and some attention-based algorithms.

Classical machine learning is currently the most widely used approach in HM risk research. Based on the technical approach employed, it can be further divided into the following technical schools: tree-based, support vector-based, statistics-based, distance-based, and linear projection-based. Among these, tree-based algorithms are the most commonly used, mainly including Decision Tree (DT), Random Forest (RF), Gradient Boosting Decision Tree (GBDT), Light Gradient Boosting Machine (LGBM), Adaptive Boosting (Ada-Boost), CatBoost, and Extreme Gradient Boosting (XGBoost). Support vector-based algorithms include Support Vector Machine (SVM) and Support Vector Regression (SVR). Statistical-based algorithms include Naive Bayes (NB) and Gaussian Process Regression (GPR). Distance-based algorithms include K-Nearest Neighbors (KNNs) and K-means Clustering (K-means). Linear projection-based algorithms include Principal Component Analysis (PCA) and Linear Discriminant Analysis (LDA).

During the literature survey, we also found several algorithms that, while not inherently machine learning algorithms, are frequently used alongside them and play a crucial role in HM risk research. They mainly fall into two categories: algorithms for feature analysis and algorithms for risk probability calculation. Algorithms for feature analysis include: SHapley Additive exPlanations (SHAP), Local Interpretable Model Agnostic Explanations (LIME), Boruta: Wrapper Algorithm for All Relevant Feature Selection (Boruta), Weighted Quantile Sum (WQS), Bayesian Kernel Machine Regression (BKMR), Restricted Cubic Spline (RCS), Cox Regression (Cox), Positive Matrix Factorization (PMF), and Structural Equation Model (SEM). Algorithms for risk probability calculation include Monte Carlo Simulation (MCS) and Bayesian Model (BM).

### 3.2. Machine Learning Algorithms

#### 3.2.1. Early Machine Learning

Early machine learning algorithms primarily include MLR, GLM, GAM, LASSO, Ridge, and Logistic, all of which belong to the generalized linear model family. Compared with classical machine learning and deep learning, early machine learning has relatively simple structures and weaker capabilities. However, due to their better interpretability and relatively lower requirements for training data, they still hold a certain position in HM risk research.

The MLR makes predictions based on a linear relationship between independent and dependent variables, requiring that the variables satisfy constraints such as linear correlation and normal distribution [211]. Applying MLR, the status or risk levels of HMs can be predicted from multiple variables [34,40,62]. Furthermore, MLR is often combined with PCA for risk source analysis [24,201].

GLM preserves the linear structure of the predictor but allows the dependent variable to follow the exponential family distribution [212]. GAM further relaxes the constraints, requiring only that the effects of each independent variable be additive. Its core idea is to fit the relationship between each independent variable and the dependent variable by adding smooth functions [213]. GLM and GAM have similar application scenarios to MLR, but they offer better capabilities for fitting nonlinear relationships among variables [66,72,98,111].

LASSO regression and Ridge regression are two important extensions of MLR, primarily used to address overfitting and multicollinearity issues. They constrain regression coefficients by adding a penalty term to the loss function through regularization. Specifically, LASSO regression adds an L1 norm penalty term (the sum of the absolute values of the coefficients) to the loss function, while Ridge regression adds an L2 norm penalty term (the sum of the squares of the coefficients) [214]. Both LASSO and Ridge regression can improve model predictions [56,71]. Furthermore, LASSO regression can automatically set the coefficients of unimportant variables to 0, allowing for model simplification and even enabling variable importance assessment and selection [46,60].

Logistic regression maps the predictions of linear combinations to the range 0 to 1 using the logistic (sigmoid) function, which represents the probability of an event occurring [215]. Despite its name, which contains “regression,” it is essentially a classification model, primarily used in HM risk research to determine risk levels or categories [36,65].

#### 3.2.2. Classical Machine Learning

Compared to early machine learning algorithms, classical machine learning algorithms are well established. They perform effectively in classification, regression, clustering, and dimensionality reduction [216]. Classical machine learning algorithms are currently the most widely applied approach in HM risk research. These algorithms can be categorized by structural and technical characteristics into support vector-based, tree-based, distance-based, statistical-based, and linear projection-based.

Support vector-based algorithms are a class of supervised learning models, with SVM being a representative example. The core idea is to find a hyperplane that not only separates samples from different classes but also maximizes the distance (called the “margin”) from the nearest points (support vectors) to the hyperplane in both classes [217]. SVM is widely used in HM risk research for tasks such as classification, feature selection, and risk-level determination [57,114,128]. SVR is a generalization of SVM for regression tasks and can be used to predict pollutant concentrations of HMs [64].

Tree-based algorithms are a well-developed class of machine learning algorithms. Their core idea is to construct a tree structure by recursively partitioning the feature space using “if–then” rules. Decision trees are the most basic algorithm; they select the optimal features for splitting based on criteria such as information entropy or the Gini coefficient, ultimately outputting either a category (classification tree) or a value (regression tree) at the leaf nodes [218,219]. However, single decision trees are prone to overfitting and exhibit weak generalization. To address this issue, ensemble learning methods combine multiple trees [220]. Random forests construct multiple independent trees by randomly sampling features and samples, and output results using voting or averaging, reducing the risk of overfitting [221]; meanwhile, gradient boosting trees (such as GBDT, AdaBoost, XGBoost, LGBM, and CatBoost) adopt a sequential strategy, where each new tree attempts to correct the prediction residuals of the previous tree, gradually improving accuracy [220]. Tree-based models naturally support nonlinear relationships and can handle mixed-type features (numerical or categorical). They perform exceptionally well on structured data and are among the most widely used machine learning approaches in HM risk research. They are widely applied in many research scenarios, including regional risk assessment [25,166], research on the pathogenicity of HMs [105,192], toxic effect research [118,188], risk source analysis [150,155], risk driver analysis [101,195], etc.

Distance-based algorithms include KNN and K-means. They make decisions based on distance or similarity metrics. KNN is primarily a supervised learning algorithm, while K-means is an unsupervised learning algorithm. KNN operates on the principle of instance-based learning, where predictions for new data points are made by leveraging the labels of their K closest training samples in the feature space [222]. K-means is a clustering algorithm. Its core idea is to partition a dataset into K compact, distinct clusters via iterative optimization [223]. Their application areas include regional risk assessment [38,124], research on the pathogenicity of HMs [96,112], risk source analysis [186,202], etc.

Statistical-based machine learning algorithms mainly include NB and GRP. They are developed based on probabilistic theory and statistical principles. These algorithms typically require datasets to follow specific statistical distributions, such as the Gaussian distribution [224,225]. They can perform tasks such as classification and regression by learning the data’s statistical distribution. One of the key advantages of these algorithms is their ability to quantify uncertainty, effectively handle small sample sizes, and incorporate prior knowledge into the learning process. They are used in research on the effects of HM stress, the pathogenicity of HMs, and the search for biomarkers associated with HMs [44,57,151].

Algorithms based on linear projection include PCA and LDA. PCA is an unsupervised dimensionality reduction and clustering method. Its basic principle is to find the principal components with the largest variance in the data, and then project the original data onto these directions via a linear transformation, thereby reducing dimensionality while preserving the main variation in the data [226]. LDA, on the other hand, is a supervised learning method. Its principle is to find the projection direction that maximizes the ratio of between-class divergence to within-class divergence, so that after projection, samples of different classes are separated as much as possible, and samples of the same class are clustered as much as possible, thus achieving feature extraction that is beneficial for classification [227]. PCA and LDA are suitable for analyzing the sources and drivers of risk [24,30,63,201].

#### 3.2.3. Deep Learning

Deep learning uses several processing layers to help computers learn and understand data in more complex ways. Its core is a multi-layer artificial neural network [228]. Deep learning transforms raw input data into higher-level, more abstract representations by layering simple nonlinear modules, enabling computers to automatically learn features at multiple levels of abstraction from the data, thereby enabling “end-to-end” learning [229]. Our investigation indicates that the application of deep learning in HM risk research remains limited. The primary algorithms employed include MLP, SOM, LSTM, RNN, GRU, CNN, and some attention-based algorithms.

MLP is a basic artificial neural network consisting of an input layer, one or more hidden layers, and an output layer. Neurons in each layer are fully connected to those in the next layer. MLP introduces nonlinear transformations through nonlinear activation functions (such as ReLU and Sigmoid) in the hidden layers, enabling it to fit nonlinear relationships [230]. Its application scenarios are similar to those of classical machine learning methods, such as SVM and RF, and it is suitable for supervised learning tasks such as classification and regression [158,160].

SOM is an unsupervised neural network model that maps input data onto a low-dimensional (usually 2D) grid via a competitive learning mechanism, preserving the topological structure of the input space. It is widely used in data clustering, feature extraction, and in HM risk research, primarily for risk source analysis [95,145].

RNNs are neural networks that excel at processing sequential data. However, traditional RNNs are prone to vanishing or exploding gradients in long sequences, making it difficult to capture long-range dependencies. LSTM and GRU are two variants designed to address this problem. LSTM, by introducing an input gate, a forget gate, an output gate, and an independent cell state, can finely control information forgetting, updating, and output, thereby effectively capturing long-range dependencies. GRU can be seen as a simplified version of LSTM, combining the forget and input gates into an update gate and fusing the cell state and hidden state, resulting in a simpler structure. In HM risk studies, they are used to predict HM concentrations at various stages of food processing [121,125]. In addition, some attention-based algorithms, such as Informer and Pyraformer, have also been applied to research on HM risks from food. Informer introduces a sparse attention mechanism to reduce computational complexity, while Pyraformer introduces a pyramid attention module to capture neighboring points and multi-scale dependencies across time [148,159].

CNNs are deep learning algorithms specifically designed for processing data with a grid-like structure, such as images. By incorporating core modules such as convolutional, pooling, and fully connected layers, they efficiently capture local spatial correlations in the data [231,232]. CNNs have become a core technology in fields such as computer vision. In HM risk research, CNNs are currently mainly used for diagnosing HM-related diseases (primarily skin diseases) [126,138].

### 3.3. Auxiliary Algorithms

Machine learning is often regarded as a black-box model due to its nonlinearity and complex feature interactions, which obscure its prediction and decision-making processes and may undermine the reliability of its outputs [233]. In addition, challenges such as feature-importance analysis, joint-effect analysis of input variables, model-uncertainty assessment, and the calculation of risk-occurrence probabilities cannot be resolved by machine learning models themselves. These issues are closely related to the identification of risk sources, risk drivers, and biomarkers associated with HMs. Consequently, a range of auxiliary machine learning algorithms has been developed. The main function of these algorithms is to analyze the relationship between model input features and output results or to calculate the probability of events occurring. They are usually used alongside machine learning models and can be categorized into two groups: those that analyze input features and interpret decision outcomes, and those that analyze risk probabilities (Figure 2).

#### 3.3.1. Algorithms for Feature Analysis

SHAP and LIME are two of the most common machine learning interpretability algorithms, primarily used to analyze the contribution of each input feature to machine learning predictions. SHAP, based on the Shapley value in game theory, assigns each feature to the prediction result. LIME perturbs the sample set around the sample to be interpreted and fits an interpretable surrogate model via a kernel-weighted summation [234]. In HM risk research, SHAP and LIME are often used to analyze the factors influencing HM exposure [188], the relationship between HMs and various diseases [93], and the sources or driving factors of risks [166,195].

Boruta is an algorithm that can identify the important features in a dataset related to a prediction target. It evaluates feature importance using a specific machine learning model (such as RF), with its core mechanism being the introduction of randomly generated “shadow features” as a benchmark. The algorithm iteratively compares the importance of real features with that of shadow features and uses statistical tests to determine whether a feature significantly outperforms random performance, thereby finding all relatively important features [235]. In HM risk research, Boruta is usually used for feature selection as input to machine learning models [35,131].

BKMR and WQS can be used to analyze the combined effects of multiple variables. BKMR uses kernel functions to automatically capture complex, nonlinear relationships between multiple factors and model predictions, as well as interactions among features, and performs parameter estimation and variable selection via a Bayesian framework based on Markov chains and Monte Carlo simulations [236]. WQS transforms multiple relevant factors into quantiles and assigns a non-negative weight to each factor via constrained optimization (usually combined with Bootstrap sampling), thereby constructing a single weighted sum of quantiles that reflects the relationship between factors and prediction results and quantifies the relative importance of each factor [237]. Their main application in HM risk research is assessing the combined effects of HMs or other risk factors [61,80,173,209].

Based on the covariance matrix of the variables, SEM analyzes causal pathways among variables by establishing measurement models between latent variables (abstract constructs that cannot be directly measured) and manifest variables (observable indicators), as well as structural models of relationships among latent variables [238]. In HM risk research, SEM can be used to integrate HM concentration data in environmental media, biological exposure indicators, and ecological or health effects to quantitatively analyze the multi-stage transmission pathways of HMs from source to exposure to risk characterization, and assess the direct and indirect impacts of each stage, thereby identifying key risk factors and their mechanisms of action [64,98,149].

PMF is a multivariate factor analysis algorithm. Its basic principle is to decompose the risk recipient data matrix into two non-negative matrices (i.e., factor contribution matrices and a factor spectrum matrix), and to solve it by minimizing the weighted least-squares objective function [239]. It is commonly used for analyzing risk sources [32,185].

The basic principle of RCS is to divide the independent variable into multiple nodes within its range, fit a cubic polynomial between each pair of adjacent nodes, and construct a smooth overall curve subject to certain constraints [240]. It can demonstrate the dose–response relationship between risk sources and risk levels, and estimate risk level changes near specific thresholds [97,98].

#### 3.3.2. Algorithms for Risk Probability Calculation

The probability of a risk event is a central concern in risk research. MCS is a widely adopted numerical method based on random sampling and is frequently employed in practical applications. This approach involves repeatedly generating large sets of random numbers that follow the probability distributions of input variables. It enables machine learning models to perform thousands of iterative calculations to estimate the probabilities of risk events [241]. In HM risk research, Monte Carlo simulation generates a large number of model input parameter samples by random sampling from the probability distributions of the risk prediction and assessment model’s input parameters. The prediction or assessment model then calculates the risk level for each sample and finally estimates the probability of risk events occurring at different risk levels [147,191,210]. Besides Monte Carlo simulation, BM are also occasionally used to calculate risk probabilities [29,62].

### 3.4. Evaluation Metrics for Machine Learning Algorithms

The performance evaluation of machine learning algorithms depends on the task type—prediction (regression) or classification—and no single metric can fully capture model performance; multiple complementary metrics are typically required.

For prediction (regression) tasks—where continuous variables such as soil heavy metal concentrations or blood lead levels are predicted—the most commonly used metrics include the coefficient of determination (R^2^), root mean square error (RMSE), and mean absolute error (MAE). R^2^ measures the proportion of variance explained by the model (ranging from 0 to 1, with higher values indicating better fit). RMSE assigns greater weight to larger errors, making it suitable for scenarios where extreme prediction deviations must be strictly controlled. MAE reflects the average absolute deviation between predicted and observed values, offering more intuitive interpretation in the original unit of measurement. A substantial discrepancy between RMSE and MAE suggests that the error distribution is uneven, with the presence of extreme outliers.

For classification tasks—where discrete categories such as contamination grades or binary health risk outcomes are predicted—commonly used metrics include accuracy, precision, recall, F1-score, and AUC-ROC. Accuracy can be misleading when classes are imbalanced. In such cases, precision (the proportion of positive predictions that are actually correct) and recall (the proportion of actual positives correctly identified) are more informative. The F1-score, the harmonic mean of precision and recall, is particularly valuable when both false positives and false negatives carry high costs, as in health risk screening, where neither missing high-risk individuals nor generating excessive false alarms is acceptable. AUC-ROC measures the model’s ability to distinguish between positive and negative classes across all classification thresholds (ranging from 0.5 to 1.0), and is relatively robust to class imbalance; it is widely regarded as one of the most informative metrics in heavy metal health risk assessment.

## 4. Results of the Survey

### 4.1. Overview of the Literature Survey

Based on 182 papers retrieved from Web of Science and Scopus, we conducted a statistical analysis of the applications of machine learning in HM risk research. The analysis covers general trends, an overview of different machine learning algorithms, and an overview of applications across different research areas. Details of each paper can be found in the Supplementary Materials (Files S1 and S2).

#### 4.1.1. General Trends

**Figure 3:**
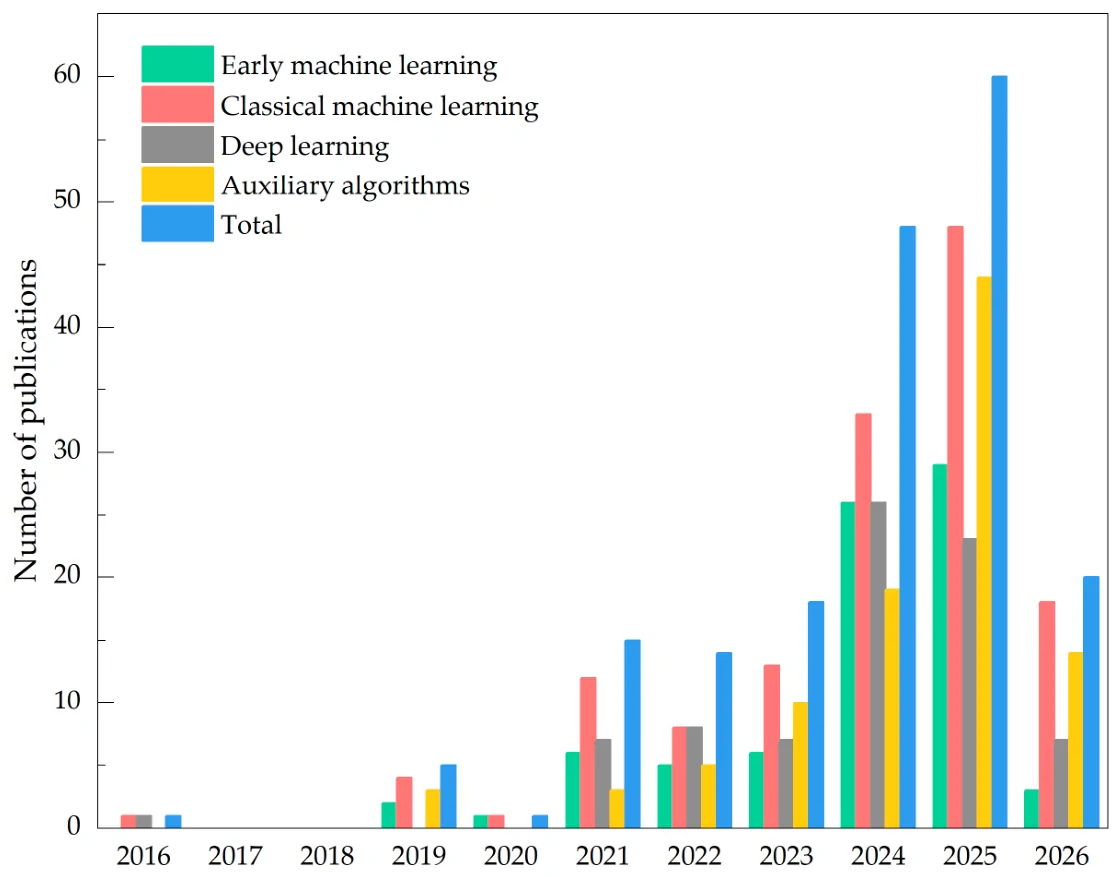
Publications on different machine learning-related algorithms used in heavy metal research across various years.

Figure 3 presents the distribution of publications related to machine learning and HM risk research over the past decade. The number of published papers has generally increased. However, this growth is not consistent across all periods. Prior to 2021, the application of machine learning in HM risk research was infrequent. Some years lacked any publications on the topic. Following 2023, the number of relevant papers increased markedly. This pattern may be influenced by the global AI boom sparked by ChatGPT’s success at the end of 2022, which stimulated interest in applying machine learning to HM research and contributed to its broader adoption in this field. The figure indicates fewer papers in 2026 because this review includes only publications released before March 2026.

Further analysis of publications on different algorithm types indicates that in most years, classical machine learning accounts for the largest share of papers. While early machine learning algorithms were not the most advanced, they still have many applications. In certain years, such as 2024 and 2025, their applications even exceeded those of deep learning. When comparing the development of auxiliary algorithm applications with that of machine learning algorithms, the number of related papers for both has generally exhibited a synchronous growth trend. This pattern suggests that scholarly attention toward auxiliary algorithms is also increasing.

The geographical distribution of research cases shows that China contributed the most (43.41%), followed by the United States at 29.12%. The two countries combined accounted for 72.53%, far exceeding other countries (See Supplementary File S2_List of reviewed papers). For China, this is mainly due to two reasons: first, industrialization has led to widespread and complex heavy metal pollution, creating an urgent need for related research; second, China’s continuous investment in environmental protection has supported the related research. For the United States, the main reason is that the National Health and Nutrition Examination Survey (NHANES) provides standardized, nationally representative data on heavy metal exposure and health indicators, which is freely available, greatly lowering the research threshold and significantly promoting the application of machine learning in the field of human health risk.

#### 4.1.2. Overview of Different Algorithms’ Applications

**Figure 4:**
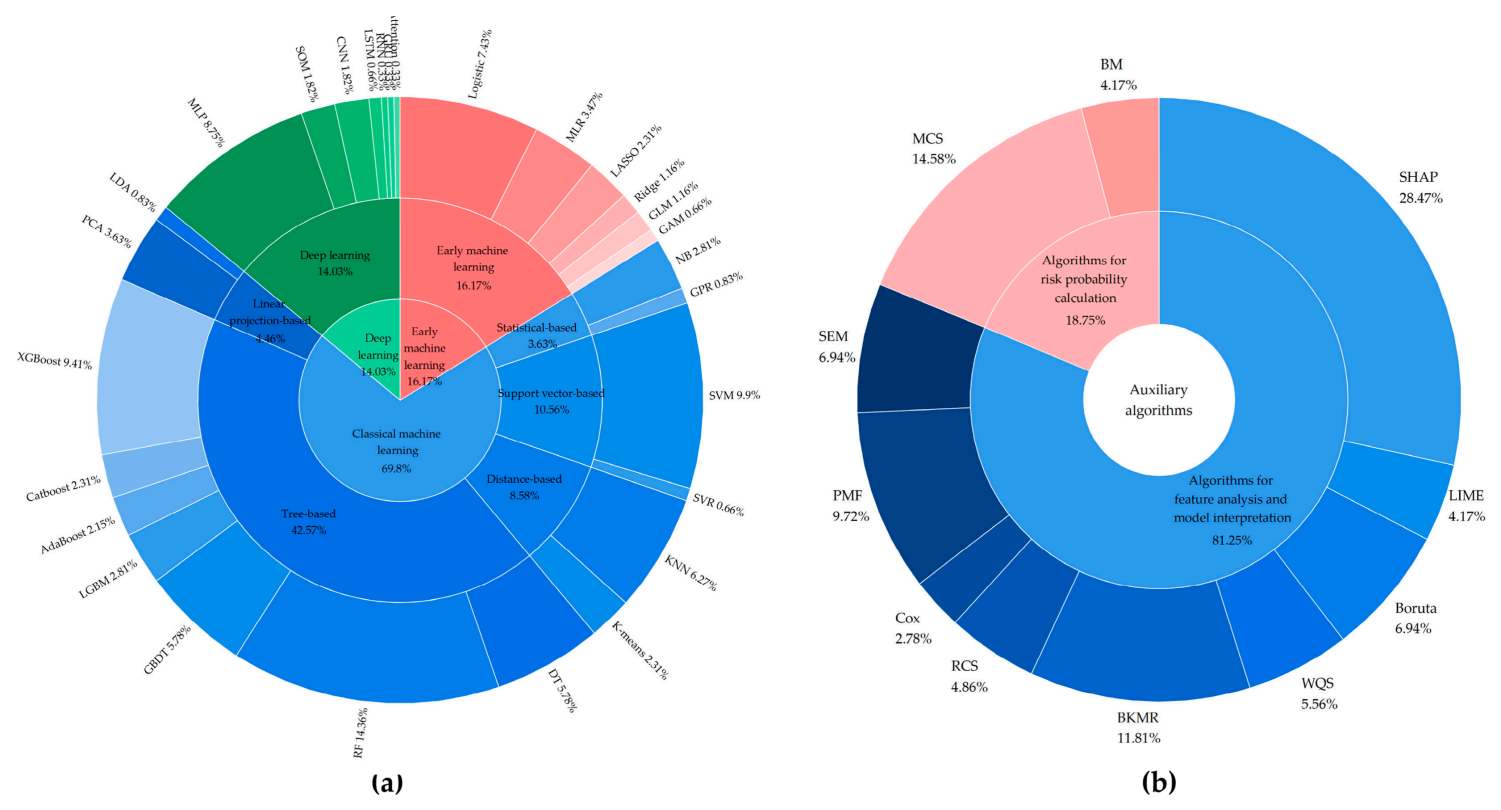
Applications of different algorithms in heavy metal risk research: (**a**) The proportion of different machine learning algorithms. (**b**) The proportion of different auxiliary algorithms.

Figure 4 illustrates the application of different machine learning algorithms and auxiliary algorithms in HM risk research. As shown in Figure 4a, classical machine learning algorithms constitute the majority at 69.8%, while deep learning has the lowest proportion (14.03%), even lower than early machine learning (16.17%). Among the different types of classical machine learning algorithms, tree-based algorithms account for the largest proportion, at 42.57%, and ensemble tree-based algorithms such as RF, GBDT, and XGBoost have become mainstream.

As shown in Figure 4b, among the different types of auxiliary algorithms, algorithms for feature analysis are the dominant approach (81.25%). This is because much of the work in HM risk research involves analyzing risk sources and driving factors, which requires algorithms capable of assessing how model input features affect prediction results [127,154]. Among these algorithms for feature analysis, SHAP has the largest share (28.47%), followed by BKMR (11.81%) and PMF (9.72%).

#### 4.1.3. Overview of Applications in Different Research Areas

**Figure 5:**
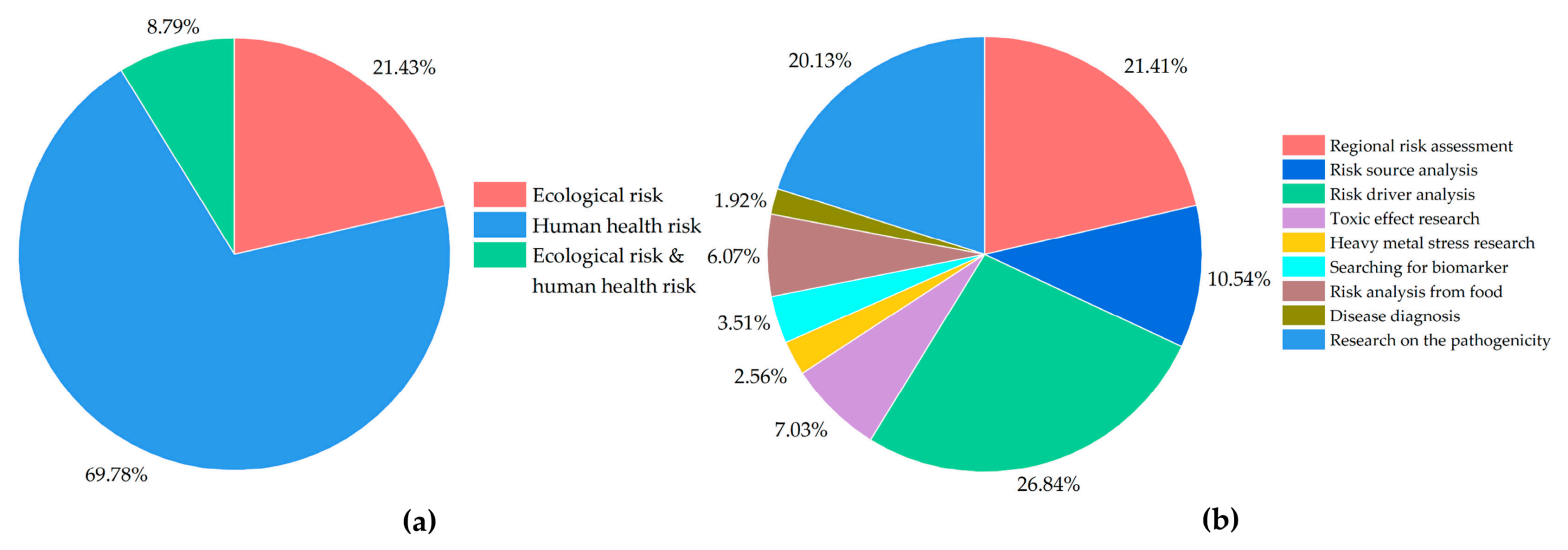
Applications of machine learning in research related to heavy metal risk: (**a**) The proportion of different types of risk research. (**b**) The proportion of different research areas.

Figure 5 illustrates the applications of machine learning in different research areas related to HM risk. Figure 5a shows that the vast majority of research cases are concentrated in human health risks, accounting for 69.78%, while ecological risk research accounts for only 21.43%, and 8.79% involve both types of risk research.

Figure 5b shows the application of machine learning in different research sub-areas. Machine learning is most commonly used in risk driver analysis (26.84%), regional risk assessment (21.41%), research on the pathogenicity (20.13%), and risk source analysis (10.54%). Collectively, these four areas comprise 78.92% of the total applications.

### 4.2. Applications of Machine Learning in Different Research Areas Related to Heavy Metal Risk

#### 4.2.1. Regional Risk Assessment

Regional risk assessment is a method used to estimate and compare environmental impacts affecting large geographical areas [242]. One of its key characteristics is the need to comprehensively consider the impacts of multiple risk factors on receptors within the region [243]. Regional risk assessments related to HMs typically determine risk levels according to ecological or human health indicators, such as the Potential Ecological Risk Index (PERI), Carcinogenic Risk index (CR), Total Carcinogenic Risk index (TCR), Hazard Index (HI), and Hazard Quotient (HQ). To calculate these indicators, it is first necessary to predict the concentration or exposure dose of HMs, which is influenced by factors such as soil, hydrology, meteorology, and bioavailability. Therefore, regional risk assessments of HM need to consider numerous factors; some studies have considered dozens of them [26,86], requiring risk assessment models capable of handling high-dimensional, nonlinear data. Traditional risk assessment methods struggle to meet this challenge, leading researchers to increasingly focus on machine learning methods [153]. Algorithms used for regional risk assessment related to HMs primarily consist of classic machine learning algorithms such as SVM, RF, XGBoost, MLP, etc. [36,94,193]. Deep learning and early machine learning algorithms, such as SOM and Logistic, also have some applications [36,127,145].

Bi et al. (2024) collected 16 factors influencing HM exposure and used various machine learning algorithms (including DT, RF, SVM, KNN, MLP, etc.) to conduct a regional risk assessment of cultivated land in Xinyang County, Hubei Province, China, based on PERI [155]. Xu et al. (2025) used machine learning algorithms such as RF, SVM, and XGBoost to predict the concentrations of 10 HMs (As, Cd, Co, Cr, Cu, Mn, Ni, Pb, Se, and Zn) in soils in northern China based on 34 environmental variables, and conducted a human health risk assessment based on CR, HI, and HQ [86]. Zhang et al. (2024) [34] used four types of data (soil properties, bioavailable HMs extraction methods, total content, and bioavailable content of HMs) and employed RF, MLP, and MLR to predict the bioavailability of seven HMs (As, Cd, Cr, Cu, Ni, Pb, and Zn) in China. Based on this, a comprehensive assessment of the ecological risks posed by HMs, with a focus on bioavailability, was conducted [34]. Shahsavani et al. (2025) [169] applied RF to predict the concentration of HMs in atmospheric particulate matter and conducted a regional health risk assessment in the area surrounding Lake Bakhtegan in Iran. The results indicate that Lifetime Cancer Risk was higher for adults than for children [169].

To improve the performance of machine learning models, some researchers have developed ensemble learning methods that rely on multiple models rather than a single model to make decisions. Hao et al. (2024) [37] constructed a multilevel ensemble learning model based on a series of DTs, using 17 environmental factors affecting HM transmission as inputs to predict HM concentrations in paddy field soil. Based on HI and TCR, they assessed the regional health risk in a typical rice-production area in Xiangtan, China [37]. Cao et al. (2025) [26] used a groundwater As dataset as the response variable and 39 environmental factors as predictor variables. Using XGBoost, RF, and SVM as base learners, they built a stacked ensemble model to predict whether groundwater As concentrations exceed health standards, thereby assessing the As health risk in shallow groundwater of the alluvial plains in the lower Yellow River, China [26]. Sakizadeh et al. (2024) used a stacking learning model (including XGBoost, RF, MLP, SVM, and GLM with Lasso or Elastic Net regularization) to forecast the spatial variation in Mn, Ca, Pb, and nitrate in the Qom-Kahak Aquifers, Iran, and to conduct a health risk assessment [110].

In addition to risk assessment indicators such as PERI, the probability of risk events is also an important indicator of risk level. Some research cases conduct regional risk assessments based on risk probability, and the main tools used include MCS and BM. Hu et al. (2019) used MCS to quantify the probabilities of exceedance of health risk guidelines due to chronic exposure to HMs and exceedance of aesthetic objectives due to elevated less toxic HMs concentrations [200]. Senoro et al. (2022) used the MCS method to evaluate the carcinogenic risk and the machine learning geo-statistical interpolation technique to create spatial maps of the metal concentrations and health risk indices [147]. Based on the relationships among water system characteristics, geographic and demographic characteristics, and children’s blood Pb concentrations, Mulhern et al. (2022) used Bayesian networks to predict the probability that children in Wake County, North Carolina, USA have blood Pb levels exceeding healthy standards, and thus assess local children’s Pb exposure risk [29].

The spatiotemporal pattern of risk depends on the spatial relationship between pollution sources and effect endpoints [244]. For this reason, spatial pattern analysis is also an important component of regional ecological risk assessment. Ejaz et al. (2025) [149] proposed a novel, integrative framework that combines SOM, PERI, HI, CR and SEM to improve the assessment and management of HM risks in soils. Using SOM, they identified complex spatial patterns of HM pollution and identified key areas requiring targeted management interventions [149]. Lyu et al. (2023) used the stacking method to combine RF, LGBM, SVM, K-NN, etc., to predict HM concentration in PM_2.5_ and analyzed the spatial risk pattern using improved land-use regression [181]. In addition, algorithms such as Geographically Weighted Regression and Kriging are often combined with machine learning for spatial risk analysis [32,175].

#### 4.2.2. Risk Source Analysis

Quantitative identification of HM sources is a key strategy for preventing and mitigating these risks. However, due to the unique migration characteristics of HMs, tracing HM contamination remains challenging [201]. Risk source analysis based on machine learning leverages pattern recognition to automatically extract potential groups from large amounts of high-dimensional data, based on linear or nonlinear relationships among the model’s input features. These grouping results are then combined with domain knowledge to analyze the sources of risk. Compared to traditional methods that relay on human experience and chemical separation identification, machine learning excels at processing high-dimensional data, capturing nonlinear relationships, and analyzing dependencies among model variables [166], and can process high-dimensional data with multiple features in a short time, greatly improving work efficiency [205]. Secondly, machine learning eliminates the subjectivity of human judgment, ensuring the objectivity and repeatability of the analysis results. Furthermore, machine learning models possess strong mixed-source decoupling capabilities, enabling quantitative calculation of the specific contribution ratios of each pollution source and avoiding the limitations of manual methods, which can only provide qualitative or semi-quantitative conclusions. The main algorithms used in risk source analysis include PMF, SOM, PCA, etc.

SOM and PMF are the most commonly used methods for analyzing the sources of HM risk. Jiao et al. (2026) [166] conducted a source analysis of HMs in soil-rose systems in Kushui, Northwest China, using both SOM and PMF. The results showed that As, Ca, Cu, and Zn in the soil were mainly attributed to household coal burning, industrial emissions, fertilization, pesticide use, and road traffic [166]. Yuan et al. (2025) [124] combined SOM, PMF, and GBDT to evaluate quantitative source identification in an old industrial city. The results showed that agricultural source (25.60%), mixed source (28.39%), natural source (13.81%), and industrial source (32.20%) were identified as the major sources of HMs [124]. Li et al. (2025) [154] introduced a machine learning-driven framework designed to characterize pollution risks, identify specific sources, and assess the source-oriented probabilistic ecological risks of HMs accumulating in surface sediments from the Kongtong section of the Jinghe River in Northwest China. By integrating SOM and PMF, they identified five major HM sources: industrial and traffic activities (33.33%), agriculture (27.21%), metal manufacturing (15.49%), natural sources (12.95%), and smelting/electroplating (11.02%) [154]. Luo et al. (2026) [170] developed a comprehensive framework integrating PMF and BLISA to apportion sources of HMs in soils from Jinqu Basin, Zhejiang Province, China. The results showed that Zn, Pb, and Cu mainly originated from a mixed source of machinery manufacturing, transportation, and agricultural activities, posing the greatest ecological and non-carcinogenic risks. Ca was primarily contributed by the paper, rubber, and plastics industries, which pose higher levels of ecological and carcinogenic risks. Hg mainly originated from chemical plants in the southwestern part of the basin [170].

PCA is also frequently used in risk source analysis. Hsieh and Chiueh. (2026) applied PCA to analyze the sources of HMs in groundwater in Yilan County, Taiwan. Their results showed that principal component 1 was dominated by Cd, In, Se, Zn, and Pb, suggesting industrial inputs, while principal component 2 was characterized by As, Cr, and Ba, reflecting a mixture of geo-genic dissolution and agricultural influence [186]. Sachchu et al. (2025) also used PCA to analyze the sources of HMs in Pangas catfish from different aquaculture areas in Bangladesh [203]. Absolute Principal Component Score-Multiple Linear Regression (APCS-MLR) is a typical receptor modeling statistical technique built upon PCA that is often used for HM risk sources analysis [24,201]. It converts standardized principal factor scores into unstandardized absolute principal factor scores, then applies multiple linear regression to quantify each principal factor’s contribution to the observed metrics.

In addition to the methods mentioned above, SEM also plays a significant role in risk source analysis. Its advantage lies in its ability to further analyze the paths of interaction between different factors and to demonstrate the spatiotemporal patterns of risk sources. Meng et al. (2024) [176] assessed the cascading effects among various HM risk factors using SEM path analysis, revealing the impact of soil and atmospheric dust in northwestern Henan Province, China, on HM accumulation in wheat grains. The results showed that Ca accumulation in wheat grains mainly originates from the soil, while Pb accumulation mainly originates from atmospheric dust [176]. Xiao et al. (2026) [123] combined SEM with PMF to analyze sources of Ca risk in the Yangtze River Delta region of China. The results showed that early traffic sources were dominant, while the proportion of agricultural sources increased later [123].

#### 4.2.3. Risk Driver Analysis

Risk driver analysis is a hot topic in HM risk research. According to our statistics, cases involving risk driver research account for the largest proportion across various research fields (Figure 5). There are numerous driving factors for HM risk, and these factors often exhibit nonlinear relationships and coupling effects [166]. A change in one factor may lead to changes in other factors or even to abrupt, nonlinear changes in risk levels. Therefore, HM risk driver analysis often involves processing high-dimensional nonlinear data.

Risk driver analysis based on machine learning leverages the nonlinear mapping and high-dimensional pattern-recognition capabilities of these algorithms to automatically learn and quantify the contributions and interactions of each factor influencing risk levels from multidimensional datasets of potential drivers [176]. Key drivers are identified through feature-importance rankings and visualization tools. Unlike traditional methods, machine learning does not presuppose a simple linear relationship between drivers and risk; instead, it uses a data-driven approach, allowing the model to discover complex correlations within the data. Its primary advantage lies in its objective feature-selection capability, which automatically ranks and selects key drivers from dozens or even hundreds of latent variables, effectively reducing the subjective biases introduced by manual analysis.

Some tree-based machine learning algorithms, such as RF, can provide importance scores for each input feature in the prediction, which can be used on their own for risk driver analysis. Chen et al. (2022) [101] used RF to assess the relative importance of factors influencing HM risk. The results indicated that the distance to the nearest industrial enterprise, agricultural chemical inputs, and industrial output were the critical factors influencing the accumulation of Cd, Cu, Zn, and Pb in the soil. For these three environmental variables, the accumulated contribution rates of Cd, Cu, Zn, and Pb were 36%, 61%, 66.56%, and 72.45%, respectively [101]. Liu et al. (2021) [99] assessed multiple algorithms (Including RF, GBDT, XGBoost et al.) and identified the optimal model for predicting childhood blood Pb levels using children’s (<5 years of age) data (*n* = 23,749) from 1991 to 2015 in Broken Hill, Australia, combined with demographic, socioeconomic, and environmental influencing factors. The results showed that Children residing within 1.0 km of the central mine area or 1.37 km of the railroad exhibited higher blood Pb levels. The year of testing demonstrated the strongest interaction with all other factors. Additionally, blood Pb levels increased more rapidly in Aboriginal children compared to non-Aboriginal children at 9–10 and 12–18 months of age [99].

To improve performance, most researchers prefer to combine machine learning algorithms with some auxiliary methods. SHAP, in particular, can quantitatively attribute HM dynamics to specific environmental factors and analyze the contributions of various factors to risk predictions [40], and thus becomes the most commonly used algorithm for risk driver analysis. In a typical agricultural pond in the Lake Taihu Basin, China, Luo et al. (2025) [40] developed a framework that integrated machine learning and SHAP analysis to quantify the contributions of risk drivers and uncover the dominant mechanisms regulating HM behavior. The characteristics of the artificial irrigation system (specifically HM concentrations in irrigation water, water level fluctuations, and nutrient concentrations) were identified as the key drivers of HM dynamics and risks [40]. By integrating XGBoost, SHAP, Boruta, and SEM algorithms, Jiao et al. (2026) identified critical features and causal pathways of HM concentrations in farmland soils and revealed ecological risks and region-specific drivers of HMs across five major urban agglomerations in China [166]. Xu et al. (2025) [86] used the SHAP approach to assess the relative quantitative contributions of environmental variables to HM risk levels. The findings underscore the substantial influence of climate factors, such as relative humidity, minimum temperature, and drought index, as well as soil and surface characteristics (such as total phosphorus and bulk density), on the HM risk level [86]. By SHAP interpretation, Li et al. (2026) found lithology, population density, precipitation, elevation and cation exchange capacity as the leading drivers of health risks related to As in soils across the Tibetan Plateau [193]; Zhang et al. (2026) identified nitrate concentration as the primary driver for HM induced health risks in drinking water distribution systems from some key cities in China [195]. Huang et al. (2024) [177] investigated the relationships between soil properties and As toxicity using machine learning and SHAP analyses. They found that soil pH, Mn/Fe oxides, and clay content were the most influential factors [177].

SEM is another algorithm frequently used for risk driver analysis. Lin et al. (2025) [64] used Pearson correlation analysis and SEM to identify the key contributors to Cd in wheat. The results showed that soil pH and cation exchange capacity were the most important factors controlling the content of bioavailable Cd, and ultimately, bioavailable Cd directly affects risk level [64]. Meng et al. (2024) [176] used machine learning to predict the spatial distributions of Pb, As, and Cd in wheat grain across northwestern Henan Province, China. Meanwhile, the cascading effects among these factors were evaluated using SEM-based path analysis. The results indicate that distances from study sites to smelting factories have cascading effects on HM accumulation in wheat grain by negatively influencing atmospheric HM deposition [176]. Ejaz et al. (2025) [149] provide a detailed quantitative evaluation of the ecological and health risks correlated with HM contamination in the industrial zones of Sialkot, Pakistan. By SEM, they identified industrial effluents and agricultural activities as the primary sources of HM contamination, with a significant positive relationship between industrial wastewater discharge and elevated levels of Cr, Pb, Cu, Cd, Ni, and Zn [149].

In addition to the algorithms mentioned above, LIME, Boruta, BKMR, WQS, and RCS are sometimes used to analyze the driving factors of HM risk. Chaurasia et al. (2025) used LIME and SHAP to analyze the driving factors of Pb poisoning risk in pregnant women and unborn babies [188]. Wang et al. (2025) explored which variables had the greatest impact on HM-related glaucoma risk using LIME and SHAP [93]. Kiełbus et al. (2026) [208] applied Boruta to analyze the drivers of cancer risk. They identified that lymph node status, tumor node metastasis stage, and elevated tumor Cu levels are key features associated with colorectal cancer [208]. Su et al. (2022) [209] used BKMR to explore the associations between exposure to PFAAs and HM mixtures and kidney function in adults, and observed significant associations between PFAAs and HM mixtures with reduced estimated glomerular filtration rate and increased odds of chronic kidney disease [209].

Some researchers also combined multiple methods to analyze risk drivers; for example, Jiang et al. (2025) used SHAP and SEM together to analyze the drivers of co-toxicity risk from HMs and micro-plastics, found that HM concentration, exposure time, and micro-plastic particle size significantly modulated the magnitude of the combined toxicity, with nanoscale micro-plastics exhibiting the most pronounced amplification effect on the combined pollution toxicity [42]. Yu et al. (2026) [35] investigated the association between exposure to mixed HMs from informal e-waste recycling and the risk of dyslexia in children living in a highly exposed community. Using SHAP and BKMR, they found that mixed-metal exposure, especially elevated levels of Cr, Ni, and Pb, significantly increases the risk of dyslexia among children residing in informal e-waste recycling areas [35]. Sakizadeh et al. (2019) [191] conducted a sequential sensitivity analysis using PCA and B-splines to examine the drivers of the health risks associated with Cr in groundwater in the southwest of Iran. The results show that the main effect factors include Cr concentration in groundwater, frequency of exposure, and water intake rate [191]. Ji et al. (2025) employed WQS and BKMR to investigate the association between exposure to mixtures of HMs and the odds of having congestive heart failure in individuals with diabetes and prediabetes [65]. Yang et al. (2025) used RCS and BKMR to examine potential associations between blood HM levels and uterine fibroids [28].

#### 4.2.4. Research on the Pathogenicity

HMs, such as As, Ca, Pb, Hg, and Cr, are pervasive environmental contaminants with well-documented toxic effects on human health [245]. Exposure to these metals has significant impacts on multiple organ systems across various life stages [246]. Epidemiological studies have established associations between HM exposure and increased risks of cardiovascular disease, renal dysfunction, neurodevelopmental impairment, and cancer [247]. The primary challenge in investigating the pathogenicity of HMs is that exposures are typically low-dose, chronic, and involve mixtures of multiple metals. The underlying mechanisms are highly complex, influenced not only by environmental or biological concentrations but also by individual characteristics, lifestyle, occupational and residential environments, and co-exposure to other chemicals. These variables collectively influence the exposure, absorption, and pathogenicity of HMs. Consequently, research in this area requires integration of diverse data sources, including medical testing, environmental monitoring, socioeconomic indicators, and demographic information. Such datasets are often high-dimensional and exhibit substantial multicollinearity, necessitating adjustment for numerous confounders. These complexities pose significant challenges for traditional analytical methods in detecting weak associations, modeling intricate interactions, and mitigating bias. Machine learning approaches provide distinct advantages in this context. Tree-based algorithms, such as RF and XGBoost, can automatically identify interactions among multiple variables and capture nonlinear exposure–response relationships, thereby facilitating the prediction and analysis of the effects of HM concentrations and other covariates on disease outcomes. Regularization techniques (such as Lasso) and shadow feature analyses (such as Boruta) are effective for managing high-dimensional collinearity and for selecting key features. Algorithms like BKMR and WQS enable analysis of the combined pathogenic effects of multiple metals or other factors. Additionally, interpretability tools such as SHAP can quantitatively assess the contributions of individual HMs and other variables to disease risk.

Zhang et al. (2024) [173] used linear mixed models to evaluate the association between HMs and lung function. In the study, BKMR and WQS were used to assess the relationship between PM_2.5_-bound HM mixtures and lung function. They found that PM_2.5_-bound HM mixtures were negatively associated with lung function indicators, with Pb, Sb, Mn, and V being the most important contributors [173]. Gao et al. (2023) [67] investigated the association between microcystins, HMs, and chronic kidney disease risk using Logistic regression. In the study, BKMR was used to investigate the combined effects of microcystins, As, and Cd on chronic kidney disease. The results showed that microcystins and As were significantly associated with increased risk of chronic kidney disease [67]. Chang et al. (2024) applied SVM and RF with the feature elimination technique to analyze the impact of Cd-induced ferroptosis on seizure severity and anxiety-like behaviors [89]. Su et al. (2022) [209] used BKMR to analyze the associations between co-exposure to PFAA and HM mixtures and renal function in a cross-sectional study of Chinese adults. They found that, in the pollutant mixtures, PFHpS, As, and Sr were the predominant contributors to decreased kidney function, and stronger associations were observed in females [209].

Due to the complexity of medical testing and surveys, coupled with privacy regulations, data acquisition has consistently been one of the biggest obstacles to the application of machine learning in the medical field. The National Health and Nutrition Examination Survey (NHANES) in the USA is a continuous, nationwide health survey project conducted by the National Center for Health Statistics (NCHS), a branch of the Centers for Disease Control and Prevention (CDC). It integrates household interviews (questionnaire data) with physical examinations at mobile testing centers, bio-sample collection (blood, urine, hair, etc.), and laboratory testing data [61]. This integrated “interview + physical examination + laboratory test” design allows researchers to simultaneously obtain demographic sociological characteristics, lifestyles, objective physical examination indicators, disease diagnostic information, and in vivo exposure levels of various environmental chemicals and nutrients in a single dataset [113]. It provides a wealth of detailed information on HM exposure (e.g., blood Pb, urinary Ca, urinary As, urinary Hb, etc.) and various chronic diseases (such as chronic kidney disease, osteoporosis, cardiovascular disease, and cancers) [70]. Researchers can download de-identified public-use documents from the CDC website without a complicated ethical approval process. This makes NHANES a popular data source for research on the pathogenicity of HMs, with numerous studies originating from it. Below are some representative cases:

Zhou et al. (2025) [105] developed a machine learning-based predictive model for heavy-metal-induced metabolic syndrome. They used Logistic regression, DT, RF, XGBoost, GBDT, CatBoost, AdaBoost, NB, KNN, and MLP to identify metabolic syndrome associated with HM exposure [105]. Li et al. (2025) [70] investigated the association between chronic HM exposure and major adverse cardiovascular events utilizing a series of machine learning algorithms (including Logistic regression, RF, XGBoost, and LGBM) combined with SHAP analysis. In addition to urine HMs concentration, the model also uses demographic and socioeconomic statistics as covariates. The results demonstrate Cd’s role as a key HM affecting angina, myocardial infarction, and heart failure [70]. Tao et al. (2021) [85] examined the independent and combined associations of 14 urinary HMs and 3 serum sex steroid hormones. MLR was used to evaluate the independent associations between metal exposure and sex hormone alterations. They concluded that exposure to HMs was associated with alterations in human sex hormones, either independently or in combination [85]. Li et al. (2025) [88] employed various machine learning-related methods to explore the potential role of Cd exposure in osteoporosis. They used MLR to assess the relationship between blood Cd levels and femoral neck bone mineral density and applied recursive feature elimination methods based on RF and SVM to identify key factors influencing the outcome [88]. You et al. (2025) implemented RF, Logistic, SVM, XGBoost, LGBM, CatBoost, NB, and MLP to assess the impact of HMs or minerals on prostate cancer, and found that blood Pb, urinary Cs, and urinary Sb were positively associated with prostate cancer risk [87]. Huang et al. (2024) [61] applied GLM to study the effects of HMs on female reproductive function and revealed that HM exposures, driven mainly by Cd and Pb, were significantly correlated with ages at menarche and natural menopause, as well as reproductive lifespan [61]. To analyze the relationship between blood Cd and stroke, Zuo and Yang (2024) developed and evaluated five machine learning algorithms, including KNN, DT, Logistic, MLP, and RF, and selected the best-performing model to predict stroke risk in US adults [27].

#### 4.2.5. Disease Diagnosis

Machine learning has been used in disease diagnosis for some time and has made progress [248], particularly in medical imaging-based diagnosis [249,250]. However, the “black box” nature of machine learning means its reasoning process remains unconvincing. Furthermore, the high cost of data acquisition and annotation limits its widespread application in disease diagnosis, resulting in few clinical applications [251]. The application of machine learning to the diagnosis of HM-related diseases primarily focuses on As-induced skin diseases, and the algorithms used are typically Convolutional Neural Networks.

Jobayer et al. (2024) [138] developed a deep-learning-based classification model that can identify As-related skin diseases. By combining Conv2D layers, self-attention mechanisms, and dropout regularization, their model achieved better performance, with 91% accuracy, 91% precision, 89% recall, and 90% F1-score [138]. Saha et al. (2024) [120] used the EfficientNetB1 deep learning model to classify As-affected skin lesions autonomously. Trained on the augmented Arsenic SkinImageBD dataset, expanded to 10,180 images, the model achieved a remarkable testing accuracy of 94.69% and an F1-score of 94.8% [120]. Emu et al. (2024) [129] constructed an image database of As-induced skin diseases to train machine learning models. The total dataset size reaches 8892. This dataset contains 4446 images collected from both non-As-affected and As-affected individuals. This dataset can help dermatologists detect diseases caused by As [129].

#### 4.2.6. Searching for Biomarker

Biomarkers are quantifiable biological markers of various physiological states or specific conditions [252]. They may also be defined as biological responses that reflect the magnitude and duration of exposure to environmental contaminants and their associated toxic effects [253]. Biomarkers can serve as early markers of the biological effects of exogenous chemicals at the molecular, cellular, tissue, and organ levels [254]. They can also serve as environmental indicators and be used to assess the subchronic effects of pollutants on organisms [255]. Currently, ecotoxicology that emphasizes molecular methods represents the predominant approach. Proteogenomics, which integrates genomic, proteomic, and transcriptomic data using bioinformatic tools, is anticipated to be the next-generation methodology [256].

The potential mechanisms of HM toxicity include disrupting normal cellular function by interfering with enzyme activity, protein folding, and ion homeostasis, and triggering oxidative stress that leads to DNA damage, cell death, and inflammation. These processes generate various biomarkers in the body [257]. In recent years, many studies have gradually shifted towards multi-omics analysis to identify potential biomarkers. However, computational complexity and data integration challenges limit the application of these methods. Machine learning, with its ability to process high-dimensional data and identify complex patterns [258], can help discover reliable biomarkers, which are crucial for research on HM risk. Using machine learning to identify and screen for HM-related biomarkers is a promising area worth exploring.

Feng et al. (2022) [137] applied machine learning to identify genetic features associated with Cd damage. To better understand why diabetic mice are more sensitive to Cd-induced toxicity, they implemented a deep learning framework based on the MLP to identify gene signatures that may predict the severity of Cd-caused renal damage. A deep learning model was developed to select optimal gene signature subsets. One MLP classification model was used to learn the dependencies between gene expression in each sample and the corresponding albuminuria-level outputs, and the other MLP model was used to rank the importance of each gene [137]. Wang et al. (2024) [98] sought to evaluate the association between As exposure and cognitive impairment and to identify potential biomarkers by developing machine learning models. In the study, RCS models were constructed to assess potential nonlinear dose–response associations. SEM was used to assess the associations between cognitive level and As exposure and investigate the mediating role of neurotransmitter alterations. Researchers further used logistic regression to quantitatively assess the risk of cognitive impairment associated with As exposure. By conducting a focused evaluation of plasma metabolites associated with various neurotransmitters, they identified a distinctive plasma neuro-metabolite profile that effectively differentiated individuals with As-induced cognitive deficits from healthy controls [98]. To explore the effects of chronic low-level Pb exposure on cognitive function and hippocampal neuronal ferroptosis, Cao et al. (2024) [180] used RF to classify related genes. A Venn diagram was subsequently created to identify the genes shared between the selected genes and those potentially related to ferroptosis. Their findings reveal significant alterations in the expression of genes associated with iron metabolism and Nrf2-dependent ferroptosis following Pb exposure [180].

In addition, Zhao et al. (2024) [46] used machine learning methods to find biomarkers of prenatal As exposure in newborn cord blood. LASSO and SVM algorithms were used to find potential biomarkers for cord blood sequencing in pregnant women exposed to As. In the end, they found that 10 genes are important biomarkers of As-exposed cord blood, associated with infiltrating immune cells, which may play important roles in As-exposed cord blood [46]. Zeng et al. (2022) [57] used machine learning to screen for potential biomarkers in urine samples from Ca-exposed subjects. They compared seven machine learning algorithms (i.e., XGBoost, Logistic, SVM, MLP, KNN, NB, and RF) and found that XGBoost and RF achieved higher accuracy and predictive performance than the others. The biomarkers identified in this study were associated with changes in amino acid and purine metabolism, as well as steroid hormone biosynthesis [57]. Singhal et al. (2022) [45] used SEM and RF to identify significant genes and pathways associated with As exposure in humans, as well as their linkages with myeloma cancer cell lines. Their study identified a unique set of 147 genes associated with As exposure and linked to cancer-related molecular mechanisms [45]. Using SVM-recursive feature elimination and RF, alongside therapeutic molecular analyses, Li et al. (2025) [88] identified critical human gene targets for Ca-induced seizures. They provide evidence of how prolonged Cd exposure disrupts iron and lipid metabolism in the brain, triggering ferroptosis in the hippocampus [88].

#### 4.2.7. Toxic Effect Research

HMs are naturally occurring elements found in the Earth’s crust and are recognized as persistent environmental pollutants. Bioaccumulation of these metals can result in a range of toxic effects across various tissues and organ systems [259]. HMs exert toxicity through various mechanisms, including the generation of reactive oxygen species, disruption of cellular processes, oxidative stress, protein and enzyme inactivation, and genomic instability, leading to organ impairment, cancer, reproductive disorders, cardiac disorders, and endocrine disruption [260]. Due to the complexity of the interactions between HMs and organisms and the diversity of influencing factors, analyzing the toxic effects of HMs is often difficult [261]. Traditional approaches for investigating toxic effects, including biochemical experiments and basic statistical analyses, which are constrained by the limited number of variables assessed and reliance on subjective assumptions, frequently leads to suboptimal outcomes [262]. Compared to traditional approaches, machine learning does not rely on subjective assumptions. It can automatically capture hidden patterns in data and identify complex relationships between various variables [263]. Combining machine learning with experiments and using machine learning to analyze experimental data often yields better results in studies of the toxic effects of HMs.

Li et al. (2024) [41] investigated soil Cd ecotoxicity using a standard single-species test animal (i.e., *Folsomia candida*) in 28 naturally Cd-contaminated soils, and the MLP was used to predict Cd ecotoxicity and accumulation in *F. candida* [41]. By integrating multiple online data sources and experimental results, Huang et al. (2024) [177] applied a GBDT model to investigate the complex associations between As toxicity thresholds and diverse soil properties. The results revealed that Mn-/Fe-ox and clay content were the most influential factors in predicting EC_10_ contribution [177]. Maarefdoust et al. (2025) [132] designed an advanced CNN model that integrates the ResNet-50 architecture with a Convolutional Block Attention Module. They used this model to classify cells under different As exposure levels and analyze the toxic effects of As on cells [132].

Experiments on toxic effects are both costly, inefficient, and are subject to various ethical and regulatory restrictions. Analyzing the combined toxicity effects of multiple substances presents additional challenges. Experiments that alter only one component at a time are insufficient for identifying combined toxicity effects; investigating these effects requires numerous experiments, and interpreting the results remains complex. Machine learning provides significant advantages for studying combined toxicity effects. Through meta-analysis, researchers can collect extensive data from published literature without conducting new experiments. Systematic analysis of these data using machine learning models substantially reduces research costs and enhances efficiency. Li et al. (2024) [190] collected EC_50_ values for the toxic effects of HMs on earthworm reproduction and the corresponding soil physicochemical properties from published papers. They then used five machine learning algorithms, RF, GBDT, XGBoost, KKNN, and SVR, to study the toxic effects of HMs on earthworm reproduction. The results showed that the relative toxicity of HMs to earthworm reproduction followed the order: Cd > Cu > Pb ≈ Zn [190]. Zhen et al. (2025) [174] combined 1520 data points from 60 toxicity experiments worldwide to study how microplastics and HMs together affect terrestrial plants. They used meta-analysis and machine learning models, including XGBoost, RF, and LGBM, to analyze the synergistic toxicity of microplastics and HMs. The results show that when plants are exposed to both microplastics and HMs, their growth and physiological functions are more strongly inhibited, and oxidative stress increases [174]. In addition, Jiang et al. (2025) further applied SHAP and SEM to analyze the effects of HM concentration, exposure time, and microplastic particle size on the magnitude of combined toxicity, finding that these factors significantly modulate the magnitude of combined toxicity, with nanoscale microplastics exhibiting the greatest amplification effect on combined pollution toxicity [42].

#### 4.2.8. Heavy Metal Stress Research

Plants subjected to HM stress will undergo changes in both their external morphology and internal structure. Detecting HM stress on plants quickly, accurately, and nondestructively helps to achieve precise management of plant growth status, but traditional chemical reagent-based detection methods are laborious, destructive, time-consuming, and costly [264]. When plants are contaminated with HMs, their internal and external structures change, altering light absorption and reflection [265]. Visible- and near-infrared, as well as hyperspectral technologies, can be used to acquire spectral information from plants. Machine learning models can be built using various types of spectral data to identify stress types and quantitatively estimate the level of HM stress. This method has the advantages of being non-destructive, rapid, and suitable for large-area monitoring, which has attracted the attention of some researchers.

Yu et al. (2021) [199] combined a proximal hyperspectral imaging system with machine learning regression methods to identify canopy characteristics that respond to tobacco plants under stress from varying concentrations of Hg. Conduct the clustering analysis using PCA on spectra of tobacco plant samples; select the effective wavelengths selected by loading of PCA and competitive adaptive reweighted sampling for discriminating canopy characteristics of tobacco plants stressed by Hg [199]. Akram et al. (2025) [183] employed PCA, the multi-trait genotype-ideotype distance index, and DT to evaluate genotype performance and identify critical stress-tolerance traits. The findings indicate that HM stress substantially decreased germination, plant height, biomass, and chlorophyll content, with the most pronounced reductions occurring under combined Ca and Ni stress [183]. Tütüncü et al. (2024) [130] integrated some machine learning algorithms to predict the impact of Cd stress on myrtle’s growth parameters. By employing MLP, RF, and XGBoost, they elucidated differences in Cd tolerance among the genotypes, accurately modeled their growth responses, and found that the white-fruited genotype exhibited greater resilience to Cd stress than the black-fruited genotype [130]. Peng et al. (2025) [43] proposed a detection method for Cu stress in oilseed rape based on hyperspectral false-color images and machine learning. The central aspect of this approach is constructing hyperspectral false-color images using Principal Component Analysis (PCA). These images capture latent physiological changes as discriminative features, thereby enabling CNNs to learn features efficiently and precisely [43].

#### 4.2.9. Risk Analysis from Food

The accumulation of HMs in food can pose a serious threat to human health [260]. Food is a significant source of HM intake, and HMs from soil, water, and the atmosphere can enter food and ultimately the human body through the food chain [266]. Each stage of food production and processing influences the accumulation and final intake of HMs; therefore, HM risk analysis of food requires consideration of all stages, making it relatively complex. Machine learning can overcome the limitations of traditional statistical methods in handling high-dimensional, nonlinear food safety data. By integrating multi-source data such as soil, water sources, crop characteristics, and the food supply chain, machine learning models can efficiently identify key links in HM pollution, providing data-driven scientific evidence for tracing the source of HM risks [176], improving the intelligence level of food safety supervision, and achieving more precise risk management.

Wu et al. (2026) [115] applied XGBoost and MLR to study the health risks associated with Ca exposure from food in a densely populated, anthropogenically impacted watershed in China. The findings indicated that soils exhibited weak alkalinity (mean pH 7.75) and Cd contamination, with 15.5% of soil samples exceeding risk thresholds. The rice Cd bioconcentration factor, soil pH, organic matter, and cation exchange capacity were identified as key predictors of both non-carcinogenic and carcinogenic risk [115]. Wang et al. (2024) [62] constructed a risk prediction model based on Bayes’ theory. The model could predict the probability of wheat grain containing Cd at levels exceeding the food safety standard, given total Cd or extractable Cd concentrations [62]. Meng et al. (2024) [176] used a boosted regression tree to examine the importance of controlling factors (including HM concentrations in atmospheric dust, bioavailable and total HM concentrations in soil, soil properties, and geospatial information) on HM accumulation in wheat grain. The cascading effects among these factors were then evaluated using SEM path analysis. Results indicate that the concentrations of Pb and As in atmospheric deposition are the primary factors influencing their accumulation in wheat grain, whereas available Cd in soil accounts for up to 56.3% of Cd accumulation in wheat grain [176]. Akash et al. (2024) [143] used the target hazard quotient and an MLP to evaluate HM contamination in fish and the safe limit for fish consumption, based on published data from 2000 to 2022. Results showed that the herbivorous fishes are less contaminated and allow greater consumption. Contrarily, the allowable intake rates of carnivorous and omnivorous fishes are lower. In addition, the safe consumption rate of saltwater fishes was lower than that of freshwater and euryhaline fishes [143].

### 4.3. The Comparison and Synthesis of Algorithms Performance

#### 4.3.1. Evaluation of Algorithm Performance in Reviewed Literature

**Figure 6:**
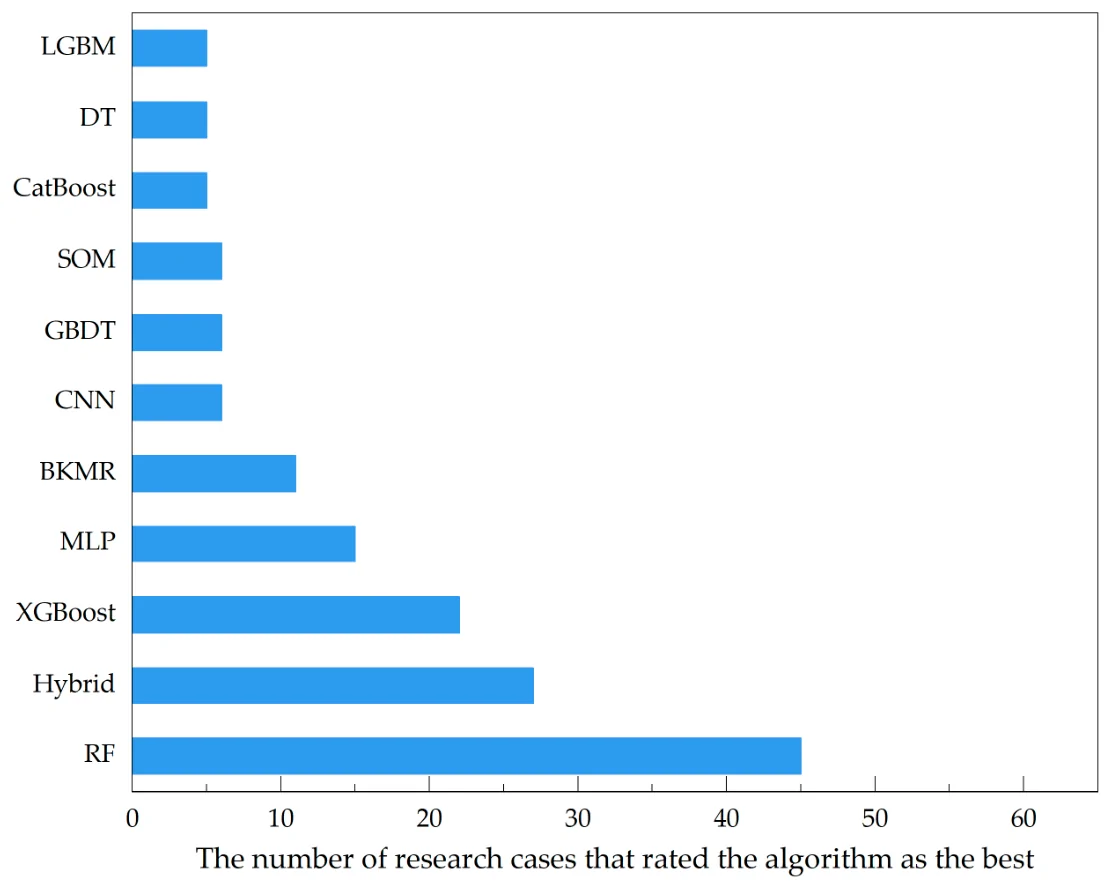
Ratings of different algorithms in various research cases.

In many studies, researchers also preliminarily evaluated and compared the performance of different machine learning algorithms. Figure 6 shows the evaluation results. RF, Hybrid, and XGBoost were rated as the best algorithms in most research cases. Among all algorithms, tree-based algorithms accounted for the largest proportion. RF and XGBoost are both tree-based algorithms; in fact, many Hybrid algorithms are composed of a series of tree-based algorithms. In addition, GBDT, CatBoost, LGBM, and DT are also tree-based algorithms. In the research cases we investigated, tree-based algorithms were rated best in 45.05% of cases. Tree-based algorithms not only have the largest proportion across different applications but also perform better.

#### 4.3.2. Synthesis of Evidence on Algorithm Performance and Applicability

Based on evidence from reviewed literature, tree-based models, especially RF and XGBoost, generally outperform other algorithms when handling tabular environmental data. However, this does not mean that tree-based models are the optimal choice in all scenarios. For example, in image recognition applications such as dermatology diagnosis, CNN have a clear advantage, and most researchers choose CNNs as their first choice. In addition, MLP perform relatively evenly across various application scenarios and therefore are favored by many researchers in some applications involving multiple tasks. Hybrid algorithms, which integrate different algorithms, can leverage the strengths of each and were rated as the optimal algorithm in the second most researched cases, second only to RF (Figure 6).

#### 4.3.3. Validation Practices and Data Limitations of Reviewed Literature

In the reviewed literature, most research cases used some form of validation procedure, such as K-fold validation. However, cases using external validation procedures are rare. This lack of external validation raises concerns about the model’s generalization ability, because models trained and tested only on the same regional dataset may capture local features of the data but cannot capture relationships between data that are generally applicable across regions.

## 5. Discussion

### 5.1. Summary of Evidence

In this review, we identified 182 case studies published over the past 10 years on the application of machine learning in HM risk research. The results show that machine learning has been widely applied in eight research areas related to HM risk: regional risk assessment, risk source analysis, risk driver analysis, research on pathogenicity, disease diagnosis, biomarker discovery, toxic effects research, HM stress research, and risk analysis in food. Commonly used machine learning algorithms include early machine learning, classical machine learning, and deep learning. Notable differences are observed in the application of machine learning across these areas and among algorithm types. Regional risk assessment, risk source analysis, risk driver analysis, and pathogenicity research of HMs are the four areas where machine learning is most widely applied. Among machine learning algorithms, classical methods dominate.

In HM risk research, classical machine learning algorithms, especially tree-based algorithms (such as RF and XGBoost), have become dominant, primarily because they are highly compatible with the characteristics of the domain’s data. These studies mainly use tabular data, which tree models are naturally suited to. Heavy metal environmental behavior is influenced by multiple factors and nonlinear interactions; tree models can automatically capture complex relationships without pre-setting interaction terms. Many studies have limited sample sizes, and tree models are more robust than deep learning with small sample sizes. Furthermore, tree models have built-in feature-importance assessment, directly identifying key driving factors, which meets the practical needs of source analysis and driving factor analysis in risk research. In addition, frameworks such as XGBoost and CatBoost further improve accuracy and generalization ability through regularization and efficient pruning. In summary, data adaptability, nonlinear modeling ability, small sample robustness, and interpretability collectively establish the dominant position of tree models in this field.

### 5.2. Challenges and Future Work Directions

#### 5.2.1. Challenges

While machine learning has been widely applied in research on HM-related risks, it still faces three core challenges.

(1) Insufficient Generalizability of Models

The inherent spatiotemporal heterogeneity of environmental processes conflicts with the fundamental assumption of many models that data are independent and identically distributed. This makes it difficult for a model trained well in one region or scale to be directly transferred to new regions with different pollution sources, geological backgrounds, or hydrological conditions, resulting in a sharp drop in predictive performance. Simultaneously, the small sample size of environmental survey data results in insufficient learning ability for rare forms or extreme events. These factors collectively constitute a severe barrier to the cross-spatial and cross-scenario generalization of machine learning models.

(2) Insufficient Reliability of Predictions

Currently, most machine learning approaches are limited to correlation-based learning and generally lack both causal inference and rigorous uncertainty quantification. Although interpretability tools such as SHAP are employed, analysis often concludes with feature-importance rankings and does not elucidate the underlying physical, chemical, or biological mechanisms that drive model predictions. This limitation has led experts in related disciplines to question the reliability of machine learning models. Additionally, most machine learning models provide only point predictions without associated confidence intervals, making it difficult to distinguish between genuine high-risk events and model errors when outcomes fall outside expected ranges. These shortcomings may restrict the use of machine learning in applications that demand high reliability, including health risk assessment, disease diagnosis, and environmental management decision-making.

(3) Difficulty in Obtaining High-quality Training Data

For HM risk research, obtaining high-precision, large-scale data is expensive, and the lack of high-quality training data is a challenge faced by many researchers. Furthermore, much of the available training data exhibits systematic limitations. First, the absence of comprehensive historical pollution emissions and hydrological dynamic data hinders models from accurately capturing the temporal accumulation of pollutants. Second, key parameters such as HM speciation and bioavailability are often missing, with only total concentration data used for training and prediction. This limitation may result in a disconnect between model predictions, evaluation outcomes, and actual biological effects.

#### 5.2.2. Future Work Directions

To address the three major bottlenecks of model generalizability, prediction reliability, and training data, future research work needs to evolve towards a more robust, reliable, and systematic approach, focusing on the following three directions.

(1) Enhancing model generalizability

Moving from purely data-driven approaches to the combination of mechanism and data-driven approaches. Future research should focus on developing hybrid models that incorporate physical and chemical laws by embedding prior knowledge, such as diffusion equations and chemical equilibrium, as constraints within neural networks, thereby limiting or normalizing the model’s extrapolation to extreme values or into new regions. Pre-training the model in a data-rich “source domain” and then transferring knowledge to the “target domain” via fine-tuning or adversarial training yields reasonable generalization. Using causal feature engineering to identify risk drivers that truly reflect environmental characteristics enables the model to learn more fundamental, time-invariant laws, thereby avoiding generalization errors caused by spurious correlations.

(2) Enhancing prediction reliability

Moving from a “correlation black box” to a “causal white box”. On the one hand, it is necessary to deeply integrate interpretability analysis with causal inference, not only identifying which features are more important but also analyzing how regulating these features affects risk levels, ensuring that the explanation conforms to scientific mechanisms. On the other hand, it is essential to promote uncertainty quantification and employ machine learning algorithms such as Bayesian neural networks that can provide confidence levels. Each prediction point should be assigned a well-calibrated confidence interval, allowing decision-makers to clearly determine whether an exceeding signal is a genuine high-risk event or a model error, thus ensuring the prediction results have the credibility to support decision-making.

(3) Overcoming data bottlenecks

Moving from passively accepting data sparsity to proactive and intelligent data acquisition. Firstly, it is necessary to integrate multi-source heterogeneous data, such as remote sensing and online monitoring, to reduce data collection costs. Secondly, proactive strategies based on model uncertainty can be adopted, allowing algorithms to guide data sampling to improve data representativeness. Furthermore, by constructing digital simulators for HM migration and transformation processes based on physical models, high-fidelity simulation data covering various historical periods can be generated to fill the systematic blind spots in historical process data, and efforts should be made to shift from predicting overall concentration to predicting risk indicators such as bioavailability, which better reflect the true risk level.

### 5.3. Contributions and Limitations

In accordance with PRISMA-ScR guidelines, this manuscript examines the application of machine learning in HM risk research, thereby improving the transparency and methodological rigor of the review. This review presents a comprehensive summary and analysis of the interdisciplinary research field, focusing not only on various machine learning algorithms but also on auxiliary algorithms closely related to machine learning that have often been overlooked in other reviews. Through systematic classification and organization, the review identifies research hotspots and knowledge gaps within the interdisciplinary domain of HM risk and machine learning. These findings offer clear directions for future research and extend the PRISMA-ScR paradigm to other disciplines.

At the same time, this review also has some limitations. First, the search scope only includes two databases: Web of Science and Scopus. While these two databases can cover much of the literature in environmental science and machine learning, they may still not be comprehensive enough. Second, PRISMA(-ScR) was originally developed for medical review reports. Its methodology may encounter challenges, such as ambiguous terminology and the absence of universally accepted standards for bias risk assessment in emerging interdisciplinary fields. Future research should investigate how to adapt PRISMA(-ScR) to diverse research paradigms, thereby supporting its evolution from a medical tool to an interdisciplinary universal framework.

## 6. Conclusions

Machine learning has been widely applied in the research of HM-related risks, covering areas such as regional risk assessment, risk source analysis, risk driver analysis, research on pathogenicity, disease diagnosis, biomarker discovery, toxic effects research, HM stress research, and risk analysis in food. The first four areas are the most frequently used, accounting for 80.06% of the total. Over the past decade, the use of machine learning in HM risk research has generally increased, especially after 2023, with the rise in a new wave of artificial intelligence. Research cases in related fields have increased significantly, accounting for 79.43% of all papers published over the past 10 years. Machine learning algorithms applied to HM risk research can be broadly divided into three categories: early machine learning, classical machine learning, and deep learning. Classical machine learning is predominant, representing 69.93% of applications, with tree-based algorithms alone accounting for 42.74% and making it the most widely used type of machine learning algorithm in HM risk research. While deep learning is a hot research topic in machine learning, its application in HM risk research is relatively limited, accounting for only 13.68%, which is even lower than early machine learning (16.39%). In addition to machine learning, auxiliary algorithms are often used in conjunction with it. Among them, algorithms for feature analysis account for the vast majority (80.58%) and are typically used to interpret machine learning model results and to analyze the sources or drivers of risk.

Currently, the application of machine learning in HM risk research faces three main challenges: insufficient model generalizability, insufficient predictive reliability, and difficulty in obtaining high-quality training data. To address these challenges, future work should focus on the following three aspects. (1) Enhancing model generalizability: Moving from purely data-driven approaches to the combination of mechanism and data-driven approaches; (2) Enhancing prediction reliability: Moving from a “correlation black box” to a “causal white box”; (3) Overcoming data bottlenecks: Moving from passively accepting data sparsity to proactive and intelligent data acquisition.

## Figures and Tables

**Table 1:** Search string for different database.

Database	Search String
Web of Science	TI = (“heavy metal*” OR “cadmium” OR “lead” OR “arsenic” OR “chromium” OR “mercury” OR “nickel” OR “copper” OR “zinc”) and TS = (“risk*” OR “stress*” OR “toxic*” OR “hazard*” OR “injur*” OR “exposure*”) and TS = (“machine learning” OR “deep learning” OR “artificial intelligence*” OR “neural network*”) and PY = (2016–2026)
Scopus	TITLE (“heavy metal*” OR “cadmium” OR “lead” OR “arsenic” OR “chromium” OR “mercury” OR “nickel” OR “copper” OR “zinc”) AND TITLE-ABS-KEY (“risk*” OR “stress*” OR “toxic*” OR “hazard*” OR “injur*” OR “exposure*”) AND TITLE-ABS-KEY (“machine learning” OR “deep learning” OR “artificial intelligence*” OR “neural network*”) AND PUBYEAR > 2015
IEEE Xplore	(“Document Title”: ”heavy metal*” OR “Document Title”: cadmium OR “Document Title”: lead OR “Document Title”: arsenic OR “Document Title”: chromium OR “Document Title”: mercury OR “Document Title”: nickel OR “Document Title”: copper OR “Document Title”: zinc) AND (“All Metadata”: risk* OR “All Metadata”: stress* OR “All Metadata”: toxic* OR “All Metadata”: hazard* OR “All Metadata”: injur* OR “All Metadata”: exposure*) AND (“All Metadata”: ”machine learning” OR “All Metadata”: deep learning OR “All Metadata”: artificial intelligence* OR “All Metadata”: neural network*) AND “Publication Year”: 2016–2026

## Data Availability

No new data were created or analyzed in this study. Data sharing is not applicable to this article.

## Supplementary Materials

The following supporting information can be downloaded at: https://www.mdpi.com/article/10.3390/toxics14080683/s1, File S1: PRISMA-ScR-Checklist; File S2: List of reviewed papers [4,6,24,25,26,27,28,29,30,32,33,34,35,36,37,38,39,40,41,42,43,44,45,46,47,48,56,57,58,59,60,61,62,63,64,65,66,67,68,69,70,71,72,73,74,75,76,77,78,79,80,81,82,83,84,85,86,87,88,89,90,91,92,93,94,95,96,97,98,99,100,101,102,103,104,105,106,107,108,109,110,111,112,113,114,115,116,117,118,119,120,121,122,123,124,125,126,127,128,129,130,131,132,133,134,135,136,137,138,139,140,141,142,143,144,145,146,147,148,149,150,151,152,153,154,155,156,157,158,159,160,161,162,163,164,165,166,167,168,169,170,171,172,173,174,175,176,177,178,179,180,181,182,183,184,185,186,187,188,189,190,191,192,193,194,195,196,197,198,199,200,201,202,203,204,205,206,207,208,209,210,267].

## Abbreviations

The following abbreviations are used in this manuscript:

AdaBoost	Adaptive Boosting
BKMR	Bayesian Kernel Machine Regression
BLISA	Bivariate Local Indicators of Spatial Association
BM	Bayesian Model
Boruta	Boruta: Wrapper Algorithm for All Relevant Feature Selection
CatBoost	Categorical Boosting
CNNs	Convolutional Neural Networks
Cox	Cox Regression
CR	Carcinogenic Risk index
DT	Decision Tree
DTPA	Diethylenetriaminepentaacetic Acid
GAM	Generalized Additive Models
GBDT	Gradient Boosting Decision Tree
GBR	Gradient Boosting Regression
GLM	Generalized Linear Model
GPR	Gaussian Process Regression
GRU	Gated Recurrent Unit
HI	Hazard Index
HM	heavy Metal
HPI	Heavy Metal Pollution Index (HPI)
HQ	Hazard Quotient
K-means	K-means Clustering
KNNs	K-Nearest Neighbors
LASSO	LASSO Regression
LCR	Lifetime Carcinogenic risk
LDA	Linear Discriminant Analysis
LGBM	Light Gradient Boosting Machine
LIME	Local Interpretable Model Agnostic Explanations
Logistic	Logistic Regression
LSTM	Long and Short-term Memory
MCS	Monte Carlo Simulation
MLP	Multilayer Perceptron
MLR	Multiple Linear Regression
NB	Naive Bayes
PCA	Principal Component Analysis
PERI	Potential Ecological Risk Index
PMF	Positive Matrix Factorization
RCS	Restricted Cubic Spline
RF	Random Forest
Ridge	Ridge Regression
RNN	Recurrent Neural Network
SEM	Structural Equation Model
SHAP	SHapley Additive exPlanations
SOM	Self-Organizing Map
SVM	Support Vector Machine
SVR	Support Vector Regression
TCR	Total Carcinogenic Risk
TRI	Target Risk Index
WQS	Weighted Quantile Sum
XGBoost	Extreme Gradient Boosting

## References

[1] Vareda J.P., Valente A.J.M., Durães L. (2019). Assessment of Heavy Metal Pollution from Anthropogenic Activities and Remediation Strategies: A Review. J. Environ. Manag..

[2] Song Y., Chen Y., Fu Z., Wen Y., Zhao W., Li J., Wang H., Du Y., Deng Y. (2025). Heavy Metal Exposure and Cognitive Impairment: An Umbrella Review of Meta-Analyses. J. Hazard. Mater..

[3] Elnabi M.K.A., Elkaliny N.E., Elyazied M.M., Azab S.H., Elkhalifa S.A., Elmasry S., Mouhamed M.S., Shalamesh E.M., Alhorieny N.A., Elaty A.E.A. (2023). Toxicity of Heavy Metals and Recent Advances in Their Removal: A Review. Toxics.

[4] Liu X., Yue F.-J., Wong W.W., Lin S.-C., Guo T.-L., Li S.-L. (2025). Arsenic Toxicity Exacerbates China’s Groundwater and Health Crisis. Environ. Int..

[5] Yi S., Li X., Chen W. (2026). An Integrated Framework Coupling Process Driven Model and Machine Learning to Simulate Soil Heavy Metal(Loid)s Accumulation and Predict Regional Risk. J. Hazard. Mater..

[6] Jiao W., Wu M., Hu T., Qi C. (2026). Global Ecological Risk Assessment of Soil Contamination by Heavy Metal(Loid)s Based on Machine Learning. J. Environ. Manag..

[7] Ray S., Vashishth R. (2024). From Water to Plate: Reviewing the Bioaccumulation of Heavy Metals in Fish and Unraveling Human Health Risks in the Food Chain. Emerg. Contam..

[8] Zhai M., Fu B., Zhai Y., Wang W., Maroney A., Keller A.A., Wang H., Chovelon J.-M. (2023). Simultaneous Removal of Pharmaceuticals and Heavy Metals from Aqueous Phase via Adsorptive Strategy: A Critical Review. Water Res..

[9] Tang J., Zhang J., Ren L., Zhou Y., Gao J., Luo L., Yang Y., Peng Q., Huang H., Chen A. (2019). Diagnosis of Soil Contamination Using Microbiological Indices: A Review on Heavy Metal Pollution. J. Environ. Manag..

[10] Bayuo J., Rwiza M.J., Choi J.W., Njau K.N., Mtei K.M. (2024). Recent and Sustainable Advances in Phytoremediation of Heavy Metals from Wastewater Using Aquatic Plant Species: Green Approach. J. Environ. Manag..

[11] Cha Z., Zhang X., Zhang K., Zhou G., Gao J., Sun S., Gao Y., Liu H. (2025). Atmospheric Heavy Metal Pollution Characteristics and Health Risk Assessment across Various Type of Cities in China. Toxics.

[12] Qin Y., Tao Y. (2022). Pollution Status of Heavy Metals and Metalloids in Chinese Lakes: Distribution, Bioaccumulation and Risk Assessment. Ecotoxicol. Environ. Saf..

[13] Kim N., Filipovic D., Bhattacharya S., Cuddapah S. (2024). Epigenetic Toxicity of Heavy Metals−Implications for Embryonic Stem Cells. Environ. Int..

[14] Mititelu M., Neacșu S.M., Busnatu Ș.S., Scafa-Udriște A., Andronic O., Lăcraru A.-E., Ioniță-Mîndrican C.-B., Lupuliasa D., Negrei C., Olteanu G. (2025). Assessing Heavy Metal Contamination in Food: Implications for Human Health and Environmental Safety. Toxics.

[15] Ghuge S.A., Nikalje G.C., Kadam U.S., Suprasanna P., Hong J.C. (2023). Comprehensive Mechanisms of Heavy Metal Toxicity in Plants, Detoxification, and Remediation. J. Hazard. Mater..

[16] Yan M., Niu C., Li X., Wang F., Jiang S., Li K., Yao Z. (2022). Heavy Metal Levels in Milk and Dairy Products and Health Risk Assessment: A Systematic Review of Studies in China. Sci. Total Environ..

[17] Kamyab H., Chelliapan S., Khalili E., Mediavilla D.P.Z., Khorami M., Aminabhavi T.M., Vasseghian Y. (2025). Nanobioremediation of Heavy Metals Using Microorganisms. J. Environ. Manag..

[18] Mallick S., Pradhan T., Das S. (2025). Bacterial Biomineralization of Heavy Metals and Its Influencing Factors for Metal Bioremediation. J. Environ. Manag..

[19] Mai X., Tang J., Tang J., Zhu X., Yang Z., Liu X., Zhuang X., Feng G., Tang L. (2025). Research Progress on the Environmental Risk Assessment and Remediation Technologies of Heavy Metal Pollution in Agricultural Soil. J. Environ. Sci..

[20] Jin L., Li C., Addou A.M., Zhang S., Li H. (2026). Global Heavy Metal-Antibiotic Co-Pollution: Distribution, ARG Co-Selection, Toxic Synergism, and AOPs-Mediated Remediation with Focus on Non-Radical Pathways. J. Hazard. Mater..

[21] Zou Y., Wang X., Khan A., Wang P., Liu Y., Alsaedi A., Hayat T., Wang X. (2016). Environmental Remediation and Application of Nanoscale Zero-Valent Iron and Its Composites for the Removal of Heavy Metal Ions: A Review. Environ. Sci. Technol..

[22] Shi J., Zhao D., Ren F., Huang L. (2023). Spatiotemporal Variation of Soil Heavy Metals in China: The Pollution Status and Risk Assessment. Sci. Total Environ..

[23] Kan X., Dong Y., Feng L., Zhou M., Hou H. (2021). Contamination and Health Risk Assessment of Heavy Metals in China’s Lead–Zinc Mine Tailings: A Meta–Analysis. Chemosphere.

[24] Chen H., Sun X., Sun L., An Y., Xiao Y., Zhang J., Hong Y., Song X. (2024). A Comprehensive Study of Spatial Distribution, Pollution Risk Assessment, and Source Apportionment of Topsoil Heavy Metals and Arsenic. Land.

[25] Li S., Yang J., Su G., Li Q., Meng J., Shi B. (2025). Analysis of heavy metal pollution characteristics and influencing factors in China’s typical agricultural areas based on Catboost algorithm. Environ. Chem..

[26] Cao W., Fu Y., Ren Y., Li X., Wang Y., Song L. (2025). Arsenic Health Risk in Shallow Groundwater of the Alluvial Plains in the Lower Yellow River, China: Driving Mechanisms of Climate Change and Human Activities. Environ. Int..

[27] Zuo W., Yang X. (2024). A Machine Learning Model Predicts Stroke Associated with Blood Cadmium Level. Sci. Rep..

[28] Yang Y., Pan M., Zhu W., Luo X., Liang X. (2025). Association between Blood Heavy Metals Exposure with Uterine Fibroids among American Women: A Cross-Sectional Analysis from NHANES Data. BMC Womens Health.

[29] Mulhern R., Roostaei J., Schwetschenau S., Pruthi T., Campbell C., Gibson J.M. (2022). A New Approach to a Legacy Concern: Evaluating Machine-Learned Bayesian Networks to Predict Childhood Lead Exposure Risk from Community Water Systems. Environ. Res..

[30] Zhao R., Lin S., Han M., Lin Z., Yu M., Zhang B., Ma L., Li D., Peng L. (2024). Association between Machine Learning-Assisted Heavy Metal Exposures and Diabetic Kidney Disease: A Cross-Sectional Survey and Mendelian Randomization Analysis. Front. Public Health.

[31] Farhadi M., Angali K.A., Sabzian M., Sepahvand A., Hayatolgheibi F., Soleimani F., Angali Z.A., Ehsani S., Behzadifar M. (2026). Heavy Metal Concentrations and Ecological Impacts (Igeo, Er, Cf, Ef, RI, and PI) in Urban Air Dust, and Those That Have the Greatest Effect on the Risk Index Evaluation Process through the Use of Machine Learning and GAM Models (Systematic Review and Meta-Analysis). Int. J. Biometeorol..

[32] Liu Z., Zeng Y., Wang W., Zhang B., Liu J., Zhao X. (2026). Coupling Positive Matrix Factorization with Machine Learning to Quantify Contamination Sources and Map Health Risks of Heavy Metals in Karst Soils. Soil Environ. Health.

[33] Yu Z., Zhang C., Xiong N., Chen F. (2021). A New Random Forest Applied to Heavy Metal Risk Assessment. Comput. Syst. Sci. Eng..

[34] Zhang J., Wang X., Li J., Luo J., Wang X., Ai S., Cheng H., Liu Z. (2024). Bioavailability (BA)-Based Risk Assessment of Soil Heavy Metals in Provinces of China through the Predictive BA-Models. J. Hazard. Mater..

[35] Yu X., Zhang X., Wen W., Lin X., Xia X., Wang D., Wu K., Huang Y. (2026). Childhood Dyslexia Risk Elevated by Heavy Metal Mixtures from E-Waste: A Machine Learning–Driven Mixture Modeling Study. Environ. Pollut..

[36] Wang D., Sun Q., Pu Y., Wang J., Xu X., Zhan M. (2025). Characteristics and Predictive Analysis of Heavy Metal Pollution in Typical Municipal Solid Waste Landfill Soil. Soil Sediment Contam. Int. J..

[37] Hao H., Li P., Jiao W., Fan H., Sang X., Sun B., Zhang B., Lv Y., Chen W., Shan Y. (2024). Environment-Compatible Heavy Metal Risk Prediction Method Created with Multilevel Ensemble Learning. J. Hazard. Mater..

[38] Li Y., Yu Y., Ding S., Dai W., Shi R., Cui G., Li X. (2025). Application of Machine Learning in Soil Heavy Metals Pollution Assessment in the Southeastern Tibetan Plateau. Sci. Rep..

[39] Wang M., Wang Y., Hu Y., Li Y. (2016). Contribution analysis of the heavy metals in the soil from different sources to the biological toxicity based on the BP neural network model. J. Saf. Environ..

[40] Luo S., Bing H., Huang J., Zhao G., Ji Y., Zhang J., Liu L., Huang Z., Gao J. (2025). Artificial Irrigation Modulates Heavy Metal Dynamics and Risks in Lowland Agricultural Ponds. J. Hazard. Mater..

[41] Li S., Li Z., Ke X., Wisawapipat W., Christie P., Wu L. (2024). Cadmium Toxicity to and Accumulation in a Soil Collembolan (Folsomia Candida): Major Factors and Prediction Using a Back-Propagation Neural Network Model. Environ. Sci. Pollut. Res..

[42] Jiang H., Che T.-H., Su Q., Jin B., Lu Y., Zhou H.-X., Fu M.-J., Zhou J.-Y. (2025). Machine Learning-Enhanced Meta-Analysis Unravels the Global Patterns of Microplastic-Heavy Metal Co-Toxicity in Terrestrial Ecosystems. Environ. Pollut..

[43] Peng Y., Sun J., Cai Z., Shi L., Wu X., Dai C., Xie Y. (2025). Copper Stress Levels Classification in Oilseed Rape Using Deep Residual Networks and Hyperspectral False-Color Images. Horticulturae.

[44] Isak M.A., Bozkurt T., Tütüncü M., Dönmez D., İzgü T., Şimşek Ö. (2024). Leveraging Machine Learning to Unravel the Impact of Cadmium Stress on Goji Berry Micropropagation. PLoS ONE.

[45] Singhal S., Ruprecht N.A., Sens D., Tavakolian K., Gardner K.L., Singhal S.K. (2022). Association between Arsenic Level, Gene Expression in Asian Population, and in Vitro Carcinogenic Bladder Tumor. Oxid. Med. Cell. Longev..

[46] Zhao X., Chen K., Wang J., Qiu Y. (2024). Analysis of Prospective Genetic Indicators for Prenatal Exposure to Arsenic in Newborn Cord Blood of Using Machine Learning. Biol. Trace Elem. Res..

[47] Lu H., Yan X., Liu S., Su Y., Yang X. (2023). Accurate characterization and health risk assessment of heavy metal pollution in soil of typical urban industrial agglomeration areas. J. Huazhong Agric. Univ..

[48] Liang C.-P., Sun C.-C., Suk H., Wang S.-W., Chen J.-S. (2021). A Machine Learning Approach for Spatial Mapping of the Health Risk Associated with Arsenic-Contaminated Groundwater in Taiwan’s Lanyang Plain. Int. J. Environ. Res. Public Health.

[49] Yuan X., Li J., Lim J.Y., Zolfaghari A., Alessi D.S., Wang Y., Wang X., Ok Y.S. (2024). Machine Learning for Heavy Metal Removal from Water: Recent Advances and Challenges. ACS EST Water.

[50] Zhao S., Guo J., Tang Y., Zhou Y. (2025). Applications of Machine Learning in Heavy Metal Adsorption Modeling: A Review. Sep. Purif. Technol..

[51] Khatoon N., Ali S., Li Z., Xue M., Han Y., Pan T., Zhang X., Yu Z., Zhao X. (2026). From Source to Prediction: Heavy Metals Toxicity, Health Risks, Detection Techniques and AI- Enhanced Predictive Models (2019–2024). J. Hazard. Mater. Adv..

[52] Moher D., Liberati A., Tetzlaff J., Altman D.G. (2010). Preferred Reporting Items for Systematic Reviews and Meta-Analyses: The PRISMA Statement. Int. J. Surg..

[53] Tricco A.C., Lillie E., Zarin W., O’Brien K.K., Colquhoun H., Levac D., Moher D., Peters M.D.J., Horsley T., Weeks L. (2018). PRISMA Extension for Scoping Reviews (PRISMA-ScR): Checklist and Explanation. Ann. Intern. Med..

[54] Jordan M.I., Mitchell T.M. (2015). Machine Learning: Trends, Perspectives, and Prospects. Science.

[55] Shi Y., Yang G., Wang X., Yu Q., Feng Y., Liu A. (2026). A Review of Deep Learning Based Anomaly Detection. Neurocomputing.

[56] Fan W., Pi Z., Kong K., Qiao H., Jin M., Chang Y., Zhang J., Li H. (2024). Analyzing the Impact of Heavy Metal Exposure on Osteoarthritis and Rheumatoid Arthritis: An Approach Based on Interpretable Machine Learning. Front. Nutr..

[57] Zeng T., Liang Y., Dai Q., Tian J., Chen J., Lei B., Yang Z., Cai Z. (2022). Application of Machine Learning Algorithms to Screen Potential Biomarkers under Cadmium Exposure Based on Human Urine Metabolic Profiles. Chin. Chem. Lett..

[58] Wu Z., Jiang S., Li J., Wang P., Chen Y. (2025). Association between Urinary Cadmium Levels and Increased Gallstone Disease in US Adults. Sci. Rep..

[59] Yang X., Li L., Nie L. (2024). Associations between Co-exposure to Heavy Metals and Vertebral Compression Fracture, as Well as Femoral Neck Bone Mineral Density: A Cross-Sectional Study from NHANES Data. PLoS ONE.

[60] Zhang Y., Li Q., Wang X. (2025). Associations between Exposure to Heavy Metal and Sarcopenia Prevalence: A Cross-Sectional Study Using NHANES Data. Front. Public Health.

[61] Huang J., Zhang Y., King L., Wang J., Nie P., Xie Q., Chen H., Wan X., Li Z., Zhao Y. (2024). Associations of Urinary Heavy Metals with Age at Menarche, Age at Menopause, and Reproductive Lifespan: A Cross-Sectional Study in U.S. Women. Ecotoxicol. Environ. Saf..

[62] Wang T., Li Y., Yang Y., Wang M., Chen W. (2024). Bayesian Risk Prediction Model: An Accessible Strategy to Predict Cadmium Contamination Risk in Wheat Grain Grown in Alkaline Soils. Environ. Pollut..

[63] Li B., Wang Q., Gui J., Yu Z., Li X., Xie C., Zhao J., Liu X., Zhang Y., Song J. (2025). Body Mass Index Mediates the Association between Heavy Metal Exposure and Overactive Bladder Risk: Insights from a Nationally Representative Cross-Sectional Study Using Explainable Machine Learning. Transl. Androl. Urol..

[64] Lin L., Zhao X., Li Y., Ling J., Ren J., Liao Q., Zhou D., Gu X. (2025). Cadmium Accumulation in Wheat Grain: Accumulation Models and Soil Thresholds for Safe Production. Eco-Environ. Health.

[65] Ji R., Wu H., Lin H., Li Y., Shi Y. (2025). Cadmium and Selenium Blood Levels in Association with Congestive Heart Failure in Diabetic and Prediabetic Patients: A Cross-Sectional Study from the National Health and Nutrition Examination Survey. Diabetol. Metab. Syndr..

[66] Angali K.A., Farhadi M., Neisi A., Cheraghian B., Ahmadi M., Takdastan A., Dargahi A., Angali Z.A. (2024). Carcinogenic and Non-Carcinogenic Risks Caused by Rice Contamination with Heavy Metals and Their Effect on the Prevalence of Cardiovascular Disease (Using Machine Learning). Food Chem. Toxicol..

[67] Gao H., Zhu N., Deng S., Du C., Tang Y., Tang P., Xu S., Liu W., Shen M., Xiao X. (2023). Combination Effect of Microcystins and Arsenic Exposures on CKD: A Case-Control Study in China. Toxins.

[68] Park H., Kim K. (2019). Comparisons among Machine Learning Models for the Prediction of Hypercholestrolemia Associated with Exposure to Lead, Mercury, and Cadmium. Int. J. Environ. Res. Public Health.

[69] Li M., Wang H., Chen Z., Liu H., Zhao H., Rong X., Xia R., Wang X., Zhou J. (2024). Contribution, Absorption Mode, and Model Prediction of Atmospheric Deposition to Copper and Lead Accumulation in Soybean. Sci. Total Environ..

[70] Li G., Zhang T., He K., Zhang M., Hu J., Ge T., Wang M., Zou R., Fan X. (2025). Deciphering the Influence of Heavy Metals on Adverse Cardiovascular Events Using Machine Learning. Toxicol. Lett..

[71] Guo T., Wang J., Meng X., Wang Y., Lou Y., Ma J., Xu S., Ni X., Jia Z., Jin L. (2024). Deciphering the Role of Zinc Homeostasis in the Tumor Microenvironment and Prognosis of Prostate Cancer. Discov. Oncol..

[72] Sengupta S., Bhattacharyya K., Mandal J., Bhattacharya P., Halder S., Pari A. (2021). Deficit Irrigation and Organic Amendments Can Reduce Dietary Arsenic Risk from Rice: Introducing Machine Learning-Based Prediction Models from Field Data. Agric. Ecosyst. Environ..

[73] Chen J. (2025). Development of a Machine Learning Model Related to Explore the Association between Heavy Metal Exposure and Alveolar Bone Loss among US Adults Utilizing SHAP: A Study Based on NHANES 2015–2018. BMC Public Health.

[74] Costa A., Sias R., Fuchs S. (2024). Effect of Whole Blood Dietary Mineral Concentrations on Erythrocytes: Selenium, Manganese, and Chromium: NHANES Data. Nutrients.

[75] Li W., Huang G., Tang N., Lu P., Jiang L., Lv J., Qin Y., Lin Y., Xu F., Lei D. (2023). Effects of Heavy Metal Exposure on Hypertension: A Machine Learning Modeling Approach. Chemosphere.

[76] Liu J., Li X., Zhu P. (2024). Effects of Various Heavy Metal Exposures on Insulin Resistance in Non-Diabetic Populations: Interpretability Analysis from Machine Learning Modeling Perspective. Biol. Trace Elem. Res..

[77] Gui Y., Gui S., Wang X., Li Y., Xu Y., Zhang J. (2024). Exploring the Relationship between Heavy Metals and Diabetic Retinopathy: A Machine Learning Modeling Approach. Sci. Rep..

[78] Nabavi A., Kashkooli M., Nabavizadeh S.S., Safari F. (2025). Heavy Metal Biomarkers and Their Impact on Hearing Loss Risk: A Machine Learning Framework Analysis. Front. Public Health.

[79] Sun T., Zheng Z., Yang M., Pan M., Tan Q., Ma Y., Zhou Y., He M., Sun Y. (2025). Heavy Metal Exposure during Pregnancy and Its Association with Adverse Birth Outcomes: A Cross-sectional Study. GeoHealth.

[80] Zhang H., Zhu X., Xin M., Wang R., Wang T., Nie G., Zhang H., Yan J., Liu C. (2025). Heavy Metal Mixtures Exposure and Breast Cancer Risk: Synergistic Effects with SHBG Identified by Mediation and Moderation Models. BMC Public Health.

[81] Jaydhar A.K., Chandra Pal S., Saha A., Islam A.R.M.T., Ruidas D. (2022). Hydrogeochemical Evaluation and Corresponding Health Risk from Elevated Arsenic and Fluoride Contamination in Recurrent Coastal Multi-Aquifers of Eastern India. J. Clean. Prod..

[82] Xia F., Li Q., Luo X., Wu J. (2022). Identification for Heavy Metals Exposure on Osteoarthritis among Aging People and Machine Learning for Prediction: A Study Based on NHANES 2011-2020. Front. Public Health.

[83] He J., Zhou W., Zhang H., Shen J. (2026). In Vivo Heavy Metal and Diabetes Association: A Cross-Sectional Interpretable Machine Learning Analysis of NHANES. Int. J. Diabetes Dev. Ctries..

[84] Chen Y., Zhao A., Li R., Kang W., Wu J., Yin Y., Tong S., Li S., Chen J. (2023). Independent and Combined Associations of Multiple-Heavy-Metal Exposure with Lung Function: A Population-Based Study in US Children. Environ. Geochem. Health.

[85] Tao C., Li Z., Fan Y., Li X., Qian H., Yu H., Xu Q., Lu C. (2021). Independent and Combined Associations of Urinary Heavy Metals Exposure and Serum Sex Hormones among Adults in NHANES 2013–2016. Environ. Pollut..

[86] Xu Y., Li P., Zhang Z., Gu Y., Xiao L., Liu X., Wang B. (2025). Integrating Machine Learning for Enhanced Spatial Prediction and Risk Assessment of Soil Heavy Metal(Loid)s. Environ. Pollut..

[87] You Z.-M., Li Y.-S., Meng F.-S., Zhang R.-X., Xie C.-X., Liang Z., Zhou J.-Y. (2025). Interpretable Machine Learning Approaches for Predicting Prostate Cancer by Using Multiple Heavy Metal Exposures Based on the Data from NHANES 2003–2018. Ecotoxicol. Environ. Saf..

[88] Li Y., Liang X., Rong Y., Jiang K., Zhang J., Li G. (2025). Investigating the Potential Risk of Cadmium Exposure on Osteoporosis: An Integrated Multi-Omics Approach. Ecotoxicol. Environ. Saf..

[89] Chang Y., Jiang X., Dou J., Xie R., Zhao W., Cao Y., Gao J., Yao F., Wu D., Mei H. (2024). Investigating the Potential Risk of Cadmium Exposure on Seizure Severity and Anxiety-like Behaviors through the Ferroptosis Pathway in Epileptic Mice: An Integrated Multi-Omics Approach. J. Hazard. Mater..

[90] Puppala S., Hossain I., Talukder S. (2023). Machine Learning and Sentiment Analysis for Predicting Environmental Lead Toxicity in Children at the ZIP Code Level. Proceedings of the 2023 IEEE 2nd International Conference on AI in Cybersecurity (ICAIC).

[91] Zibibula Y., Tayier G., Maimaiti A., Liu T., Lu J. (2024). Machine Learning Approaches to Identify the Link between Heavy Metal Exposure and Ischemic Stroke Using the US NHANES Data from 2003 to 2018. Front. Public Health.

[92] Xia T., Han K. (2025). Machine Learning Prediction Model with Shap Interpretation for Chronic Bronchitis Risk Assessment Based on Heavy Metal Exposure: A Nationally Representative Study. BMC Pulm. Med..

[93] Wang X., Chen G., He R., Gao Y., Lu J., Xu T., Liu H., Jiang Z. (2025). Machine Learning Prediction of Glaucoma by Heavy Metal Exposure: Results from the National Health and Nutrition Examination Survey 2005 to 2008. Sci. Rep..

[94] Song X., Lu R., Kuang M., Feng L., Wang Y., Wu D., Cai M., Feng Y. (2024). Machine Learning-Assisted Risk Evaluation of Heavy Metals in the Hainan Gold Mining Region, China. Environ. Monit. Assess..

[95] Li J., Li X., Tai X.-S., Tuo X.-Y., Zhou F.-Y., Rong Y.-J., Zang F. (2025). Machine Learning-Assisted Source Identification and Probabilistic Ecological-Health Risk Assessment of Heavy Metal(Loid)s in Urban Park Soils. Sci. Rep..

[96] Kim K., Park H. (2021). Machine-Learning Models Predicting Osteoarthritis Associated with the Lead Blood Level. Environ. Sci. Pollut. Res..

[97] Wu Y., Li G., Dong M., Deng Y., Zhao Z., Zhou J., Xian S., Yang L., Yi M., Yang J. (2025). Metabolomic Machine Learning Predictor for Arsenic-Associated Hypertension Risk in Male Workers. J. Pharm. Biomed. Anal..

[98] Wang W., Sun B., Luo D., Chen X., Yao M., Zhang A. (2024). Neurotransmitter Metabolism in Arsenic Exposure-Induced Cognitive Impairment: Emerging Insights and Predictive Implications. Environ. Sci. Technol..

[99] Liu X., Taylor M.P., Aelion C.M., Dong C. (2021). Novel Application of Machine Learning Algorithms and Model-Agnostic Methods to Identify Factors Influencing Childhood Blood Lead Levels. Environ. Sci. Technol..

[100] Dey A., Das Sharma K., Sanyal T., Bhattacharjee P., Bhattacharjee P. (2019). Population Based Study on Arsenic Induced Blood Samples Employing Hybrid Metaheuristic Optimization Based ML Approach. Proceedings of the 2019 IEEE Region 10 Symposium (TENSYMP).

[101] Chen H., Wang L., Hu B., Xu J., Liu X. (2022). Potential Driving Forces and Probabilistic Health Risks of Heavy Metal Accumulation in the Soils from an E-Waste Area, Southeast China. Chemosphere.

[102] Wang X., Bakulski K.M., Mukherjee B., Hu H., Park S.K. (2023). Predicting Cumulative Lead (Pb) Exposure Using the Super Learner Algorithm. Chemosphere.

[103] Shen M., Zhang Y., Zhan R., Du T., Shen P., Lu X., Liu S., Guo R., Shen X. (2025). Predicting the Risk of Cardiovascular Disease in Adults Exposed to Heavy Metals: Interpretable Machine Learning. Ecotoxicol. Environ. Saf..

[104] Rashidmayvan M., Mansoori A., Aghasizadeh M., Dianati M., Barati S., Sahranavard T., Darroudi S., Ahari R.K., Esmaily H., Ferns G. (2024). Prediction of Cardiovascular Disease Risk by Serum Zinc and Copper Concentrations and Anthropometric Measurements. J. Trace Elem. Med. Biol..

[105] Zhou J.-X., Zheng Z.-Y., Peng Z.-X., Yang Y.-T., Ni H.-G. (2025). Predictive Model in Silicon and Pathogenicity Mechanism of Metabolic Syndrome: Impacts of Heavy Metal Exposure. J. Environ. Manag..

[106] Michael T., Kohn E., Daniel S., Hazan A., Berkovitch M., Brik A., Hochwald O., Borenstein-Levin L., Betser M., Moskovich M. (2022). Prenatal Exposure to Heavy Metal Mixtures and Anthropometric Birth Outcomes: A Cross-Sectional Study. Environ. Health.

[107] Monaco A., Lacalamita A., Amoroso N., D’Orta A., Del Buono A., Di Tuoro F., Tangaro S., Galeandro A.I., Bellotti R. (2021). Random Forests Highlight the Combined Effect of Environmental Heavy Metals Exposure and Genetic Damages for Cardiovascular Diseases. Appl. Sci..

[108] Mansoori A., Ghiasi Hafezi S., Ansari A., Arab Yousefabadi S., Kolahi Ahari R., Darroudi S., Eshaghnezhad M., Ferns G., Ghayour-Mobarhan M., Esmaily H. (2025). Serum Zinc and Copper Concentrations and Dyslipidemia as Risk Factors of Cardiovascular Disease in Adults: Data Mining Techniques. Biol. Trace Elem. Res..

[109] Kim I.-G., Hong S., Yim S., Jeong J.-H., Choi K., Lee J.-H., Hong Y.-S., Eom S.-Y., Kim H., Kim Y.-D. (2024). Sex-Specific Effects of Combined Heavy Metal Exposure on Blood Pressure: A Bayesian Kernel Machine Regression Analysis. Atmosphere.

[110] Sakizadeh M., Zhang C., Milewski A. (2024). Spatial Distribution Pattern and Health Risk of Groundwater Contamination by Cadmium, Manganese, Lead and Nitrate in Groundwater of an Arid Area. Environ. Geochem. Health.

[111] Liu S., Wang H., Cao Y., Lu L., Wu Y., Lian F., Yang J., Song Q. (2025). The Association between Low-Concentration Heavy Metal Exposure and Chronic Kidney Disease Risk through α-Klotho. Sci. Rep..

[112] Angali K.A., Farhadi M., Neisi A., Cheraghian B., Ahmadi M., Takdastan A., Dargahi A. (2025). The Effect of Consuming Bread Contaminated with Heavy Metals on Cardiovascular Disease and Calculating Its Risk Assessment. Sci. Rep..

[113] Xiao H., Liang X., Li H., Chen X., Li Y. (2024). Trends in the Prevalence of Osteoporosis and Effects of Heavy Metal Exposure Using Interpretable Machine Learning. Ecotoxicol. Environ. Saf..

[114] Charan K., Ghosh S., Prajapati J., Mondal G., Bhattacharyya P. (2025). Understanding the Geochemical Footprints of Red Mud Disposal to the Neighboring Environment: A Data-Driven Case Study through Risk Assessment, Sensitivity Analysis and Source Apportionment of Heavy Metals. J. Environ. Chem. Eng..

[115] Wu Q., Hu W., Zhang S., Wang X., Fan Y., Tian K., Holm P.E., Hansen H.C.B., Huang B. (2026). Unveiling the Fate of Heavy Metals along the Soil–Rice–Human Pathway: Source–Sink Quantification, Rhizospheric Processes, and Health Implications. J. Hazard. Mater..

[116] Pan S., Niu Y., Duan S., Zhao D., Wang Q., Dong Z., Cai G., Chen X. (2024). Uric Acid Mediates the Relationship between Mixed Heavy Metal Exposure and Renal Function in Older Adult People. Front. Public Health.

[117] Gu X., Li Q., Wang X. (2025). Using Life’s Essential 8 and Heavy Metal Exposure to Determine Infertility Risk in American Women: A Machine Learning Prediction Model Based on the SHAP Method. Front. Endocrinol..

[118] Potash E., Ghani R., Walsh J., Jorgensen E., Lohff C., Prachand N., Mansour R. (2020). Validation of a Machine Learning Model to Predict Childhood Lead Poisoning. JAMA Netw. Open.

[119] Mamun A.A., Hossen M.K., Haque M.I., Ali M.M., Choudhury A.A., Uddin J. Groundwater Arsenic Contamination Prediction Using an Optimized Ensemble Model with Explainable AI. Proceedings of the 2025 28th International Conference on Computer and Information Technology (ICCIT).

[120] Saha A., Das U., Rahman M.S. (2024). A Deep Learning Approach to Automate Classification of Arsenic-Affected Skin Using EfficientNet-B1. Proceedings of the 2024 27th International Conference on Computer and Information Technology (ICCIT).

[121] Jin X., Zhang J., Wang X., Zhang X., Guo T., Shi C., Su T., Kong J., Bai Y. (2021). A Deep Network Prediction Model for Heavy Metal Cadmium in the Rice Supply Chain. J. Future Foods.

[122] Andleeb S., Bao S., Ahmad Z., Kumar Jha S. (2021). A Hybrid Machine Learning Approach in Modeling the Impact of Chromium Concentration in Blood and Gonads on the Concentration of the Reproductive Hormones of Urva Auropunctatus. Measurement.

[123] Xiao L., Feng P., Liang Y., Xia B., Ye Y., Liu H., Zhao W., Li Z., Cheng K., Chen J. (2026). A Machine Learning Framework Bridging Heavy Metal Contamination and Human Health Risks in Agricultural Soils of the Yangtze River Delta (2001–2030). J. Hazard. Mater..

[124] Yuan L., Yang Q., Ji H., Li M., Gu Q. (2025). A Novel Hybrid Approach for Quantitative Source Apportionment and Source-Oriented Health Risk of Heavy Metal(Loid)s Pollution in an Old Industrial City. J. Hazard. Mater..

[125] Wang Z., Wu Z., Zou M., Wen X., Wang Z., Li Y., Zhang Q. (2022). A Voting-Based Ensemble Deep Learning Method Focused on Multi-Step Prediction of Food Safety Risk Levels: Applications in Hazard Analysis of Heavy Metals in Grain Processing Products. Foods.

[126] Ergün E., Okumuş H. (2026). Advanced Deep Learning for Early Diagnosis of Arsenic-Induced Dermatological Conditions through Dermoscopic Image Evaluation. J. Med. Eng. Technol..

[127] Li J., Ma L.-B., Liu J.-Z., Li X., Yang Y.-Q., Tai X.-S., Zhou F.-Y., Zang F. (2025). Advanced Matter-Element Extension Model and Machine Learning for Source-Specific Probabilistic Health Risk Assessment of Heavy Metals/Metaloids in Soil-Rose Systems in Kushui, Northwest China. Ecol. Indic..

[128] Deng P., Hu X., Mu L., Yu F., Luo L. (2025). Application of a Machine Learning-Based Food Risk Framework to Assess the Public Dietary Risk of Cadmium in China. Environ. Pollut..

[129] Emu I.A., Niloy N.T., Karim B.M.A., Chowdhury A., Johora F.T., Hasan M., Mittra T., Rashid M.R.A., Jabid T., Islam M. (2024). ArsenicSkinImageBD: A Comprehensive Image Dataset to Classify Affected and Healthy Skin of Arsenic-Affected People. Data Brief.

[130] Tütüncü M., Isak M.A., İzgü T., Dönmez D., Kaçar Y.A., Şimşek Ö. (2024). Assessing Cadmium Stress Resilience in Myrtle Genotypes Using Machine Learning Predictive Models: A Comparative in Vitro Analysis. Horticulturae.

[131] Huang W., Liu D., Liu G., Chen G. (2026). Association of Urinary Heavy Metals with Osteoporosis in US Adults Using Interpretable Machine Learning. Toxicol. Lett..

[132] Maarefdoust R., Currie D., MacLeod B., Song Y., Concannon J., Frankel S., Zhang X., Su R., Frangi A., Zhang Y. (2025). Attention-Based CNN for Enhanced Detection of Arsenic Exposure. Proceedings of the 2024 International Conference on Medical Imaging and Computer-Aided Diagnosis (MICAD 2024).

[133] Chaurasia P., McClean S.I., Mahdi A.A., Yogarajah P., Ansari J.A., Kunwar S., Ahmad M.K. (2023). Automated Lead Toxicity Prediction Using Computational Modelling Framework. Health Inf. Sci. Syst..

[134] Liu M., Chen G., Wen Y., Wu L., Wang T. (2024). Combining Radiative Transfer Model with Statistical Model for Distinguishing Heavy Metal Stress Level in Rice from Sentinel-2 Satellite Images. J. Appl. Remote Sens..

[135] Maurya R., Srivastava A., Srivastava A., Pathak V.K., Dutta M.K. (2023). Computer Aided Detection of Mercury Heavy Metal Intoxicated Fish: An Application of Machine Vision and Artificial Intelligence Technique. Multimed. Tools Appl..

[136] Li P., Hao H., Bai Y., Li Y., Mao X., Xu J., Liu M., Lv Y., Chen W., Ge D. (2022). Convolutional Neural Networks-Based Health Risk Modelling of Some Heavy Metals in a Soil-Rice System. Sci. Total Environ..

[137] Feng X., Jin X., Zhou R., Jiang Q., Wang Y., Zhang X., Shang K., Zhang J., Yu C., Shou J. (2022). Deep Learning Approach Identified a Gene Signature Predictive of the Severity of Renal Damage Caused by Chronic Cadmium Accumulation. J. Hazard. Mater..

[138] Jobayer S.H., Chowdhury M.M., Siam A.S.M., Mim U.H., Zidan M.M.H.K., Bithe M.A., Hasan M.M. (2024). Deep Learning for Identification of Skin Diseases: Emphasizing on Arsenic-Induced Skin Conditions. Proceedings of the 2024 27th International Conference on Computer and Information Technology (ICCIT).

[139] Saygın F. (2024). Determination of Heavy Metal Concentrations in Cultivated Soils and Prediction of Pollution Risk Indices Using the ANN Approach. Rendiconti Lincei Sci. Fis. Nat..

[140] Nabavi A., Safari F., Kashkooli M., Sadat Nabavizadeh S., Molavi Vardanjani H. (2024). Early Prediction of Cognitive Impairment in Adults Aged 20 Years and Older Using Machine Learning and Biomarkers of Heavy Metal Exposure. Curr. Res. Toxicol..

[141] Sheng X., Shi W., Zheng J., Ma Y. (2024). Evaluation and prediction of heavy metal health risk in soils left over from an electroplating plant in zhejiang province based on BP neural network and inverse-distance weighting. Asian J. Cotoxicol..

[142] Irandoost F., Agah H., Eslami Z., Rossi L., Colloca F., Khalili A., Costantini M.L. (2021). Evaluation of Nitrogen and Heavy Metal Pollution in Southern Caspian Sea: Risk Assessment and Modeling Approach. Mar. Pollut. Bull..

[143] Akash P.B., Kumar S., Jahan M.S., Rahman M.S., Seddiky M.A., Sorker A., Islam R. (2024). Exploring Potential Human Health Risks Linked to Heavy Metal(Loid)s in Dietary Fishes: Utilizing Data-Driven and Computational Modelling Approaches. Biol. Trace Elem. Res..

[144] Li Y., Tao R., Feng Z., Mei G., Liu Z., Yang W., Mo F., Liu Z. (2025). Exploring the Association between Heavy Metal Exposure and Periodontitis Using Interpretable Machine Learning Models: NHANES 2009–2014. Hum. Ecol. Risk Assess. Int. J..

[145] Zhao D., Wang C., Zhou X., Wang R., Guo Z., Ding M., Yu B., Wang B. (2026). Human Health Risk Assessment of Heavy Metal(Loid)s in Topsoil and Groundwater from a Typical Heavy-duty Enterprise Gathering Area of China Using Self-organizing Feature Map. Water Environ. Res..

[146] Weerasinghe D., Kumara B.T.G.S., Kuhaneswaran B., Gunathilake S.K. (2021). Identifying the Type of Chronic Kidney Disease Based on Heavy Metals in Soil Using ANN. Proceedings of the 2021 Third International Sustainability and Resilience Conference: Climate Change.

[147] Senoro D.B., De Jesus K.L.M., Nolos R.C., Lamac M.R.L., Deseo K.M., Tabelin C.B. (2022). In Situ Measurements of Domestic Water Quality and Health Risks by Elevated Concentration of Heavy Metals and Metalloids Using Monte Carlo and MLGI Methods. Toxics.

[148] Lu P., Dong W., Jiang T., Liu T., Hu T., Zhang Q. (2023). Informer-Based Safety Risk Prediction of Heavy Metals in Rice in China. Foods.

[149] Ejaz U., Khan S.M., Shah S.F.A., Khalid N., Jehangir S., Rizvi Z.F., Svenning J.-C. (2025). Integrative Data-Driven Analytics for Assessing Ecological and Human Health Risks of Soil Heavy Metal Contamination. J. Hazard. Mater. Adv..

[150] Du G., Zhang Y., Zou T., Liu C., Hou Y., Li J., Deng F., An J., Feng L., Wu B. (2025). Machine Learning and Network Toxicology Reveal Arsenic as a Key Driver of Non-Carcinogenic Health Risks from Heavy Metal Residues in Chinese Medicinal Plants. Sci. Total Environ..

[151] Gao X., Liu C., Yin L., Wang A., Li J., Gao Z. (2024). Machine Learning Model for Age-Related Macular Degeneration Based on Heavy Metals: The National Health and Nutrition Examination Survey 2005 to 2008. Sci. Rep..

[152] Xia F., Li Q., Luo X., Wu J. (2022). Machine Learning Model for Depression Based on Heavy Metals among Aging People: A Study with National Health and Nutrition Examination Survey 2017–2018. Front. Public Health.

[153] Wang S., Li G., Ji X., Wang Y., Xu B., Tang J., Guo C. (2024). Machine Learning-Driven Assessment of Heavy Metal Contamination in the Impounded Lakes of China’s South-to-north Water Diversion Project: Identifying Spatiotemporal Patterns and Ecological Risks. J. Hazard. Mater..

[154] Li J., Wang C., Tuo X.-Y., Proshad R., Liu J.-Z., Gao Z.-D., Zhou F.-Y., Zang F. (2025). Machine Learning-Driven Multi-Technique Source Tracing and Source-Specific Probabilistic Ecological Risk Assessment of Heavy Metal(Loid)s in Urban River Sediments. Ecol. Indic..

[155] Bi Z., Sun J., Xie Y., Gu Y., Zhang H., Zheng B., Ou R., Liu G., Li L., Peng X. (2024). Machine Learning-Driven Source Identification and Ecological Risk Prediction of Heavy Metal Pollution in Cultivated Soils. J. Hazard. Mater..

[156] Kim K., Park H. (2024). Neural Network Approach to Predict the Association between Blood Cadmium Levels and Hypertension. Edelweiss Appl. Sci. Technol..

[157] Hu F., Jiang L., Chen Q.-H., He B.-N. (2025). pollution characteristics, source analysis, and health risk assessment of heavy metals of soil in a certain coal mining area of western jiangxi province. Huan Jing Ke Xue Huanjing Kexue.

[158] Zhang Z., Lou S., Liu S., Zhou X., Zhou F., Yang Z., Chen S., Zou Y., Radnaeva L.D., Nikitina E. (2024). Potential Risk Assessment and Occurrence Characteristic of Heavy Metals Based on Artificial Neural Network Model along the Yangtze River Estuary, China. Environ. Sci. Pollut. Res..

[159] Dong W., Hu T., Zhang Q., Deng F., Wang M., Kong J., Dai Y. (2023). Prediction of Food Safety Risk Level of Wheat in China Based on Pyraformer Neural Network Model for Heavy Metal Contamination. Foods.

[160] Gui H., Yang Q., Lu X., Wang H., Gu Q., Martín J.D. (2023). Spatial Distribution, Contamination Characteristics and Ecological-Health Risk Assessment of Toxic Heavy Metals in Soils near a Smelting Area. Environ. Res..

[161] Xu S., Sun M. (2025). The Interpretable Machine Learning Model for Depression Associated with Heavy Metals via EMR Mining Method. Sci. Rep..

[162] Xiang Q., Yu H., Chu H., Hu M., Xu T., Xu X., He Z. (2022). The Potential Ecological Risk Assessment of Soil Heavy Metals Using Self-Organizing Map. Sci. Total Environ..

[163] Antor M.S.U.M., Podder N.K., Aman N., Haque M.R., Arjon S.S., Yasmin T. Comparative Deep Learning and Explainable AI Approaches for Automated Detection of Arsenic Skin Lesions. Proceedings of the 2025 IEEE 2nd International Conference on Computing, Applications and Systems (COMPAS).

[164] Moenks N., Penava P., Buettner R. (2025). A Novel High-Performance Deep-Learning-Based Approach for the Detection of Arsenic Poisoning. IEEE Access.

[165] Qu W., Wang J., Wang K., Sun S., Zheng Y., Ji Y. (2024). Research on Real-Time Monitoring and Risk Assessment System for Ocean Heavy Metal Pollution Driven by Deep Learning. Proceedings of the 2024 International Conference on Computing, Robotics and System Sciences (ICRSS).

[166] Jiao L., Huo L., Stein A., An Y., Zhang P. (2026). A Framework for Unveiling Multi-Pathway Mechanisms of Soil Heavy Metal Pollution: Evidence from Five Major Urban Agglomerations in China. J. Environ. Manag..

[167] Singh A., Gupta H., Srivastava A., Srivastava A., Joshi R.C., Dutta M.K. (2021). A Novel Pilot Study on Imaging-based Identification of Fish Exposed to Heavy Metal (Hg) Contamination. J. Food Process. Preserv..

[168] Wang Y., Zhang Z., Liang C., Huang H., Wu K., Zhao H., Shangguan Y. (2026). A Novel Quantitative Approach for Factor Identification and Risk Prediction of Cadmium Accumulation in Wheat Using Machine Learning and Bayesian Models. Plant Soil.

[169] Shahsavani S., Shamsedini N., Mohammadpour A., Hoseini M. (2025). Air Quality near Middle East’s Large Dried Lake: Heavy Metal Emissions, Machine Learning Analysis, and Health Risks. Phys. Chem. Earth Parts ABC.

[170] Luo Q., Jia Z., Zhang F., Shi X., Xie X., Yu H., Zheng C., Zhu T. (2026). An Integrated Framework for Revealing the Impacts of Industrial Activities on Heavy Metal(Loid)s Sources and Risks in Agricultural Soils. J. Environ. Chem. Eng..

[171] Ximenez J.P.B., Zamarioli A., Kacena M.A., Barbosa R.M., Barbosa F. (2021). Association of Urinary and Blood Concentrations of Heavy Metals with Measures of Bone Mineral Density Loss: A Data Mining Approach with the Results from the National Health and Nutrition Examination Survey. Biol. Trace Elem. Res..

[172] Yuting Y., Shan D. (2025). Associations between Urinary and Blood Heavy Metal Exposure and Heart Failure in Elderly Adults: Insights from an Interpretable Machine Learning Model Based on NHANES (2003–2020). Int. J. Cardiol. Cardiovasc. Risk Prev..

[173] Zhang J., Hu Y., Wang X., Ding X., Cen X., Wang B., Yang S., Ye Z., Qiu W., Chen W. (2024). Associations of Personal PM2.5-Bound Heavy Metals and Heavy Metal Mixture with Lung Function: Results from a Panel Study in Chinese Urban Residents. Chemosphere.

[174] Zhen Y.-Q., Che T.-H., Song Y.-X., Wan Y.-M., Jiang H.-H., Yu Z.-Z., Yu M., Xu S.-Q., Li C.-Y., Zhou J.-Y. (2025). Combined Toxicity of Microplastics and Heavy Metals to Terrestrial Plants: A Global Meta-Analysis Integrating Machine Learning Predictions. J. Environ. Chem. Eng..

[175] Ding W.Q., Sawut R., Rixit G., Xu M. (2025). Combining Random Forest and XGBoost Models for Source Apportionment and Health Risk Assessments of Heavy Metals in Suburban Farmland Soils. Ecotoxicol. Environ. Saf..

[176] Meng L., Sheng A., Cao L., Li M., Zheng G., Li S., Chen J., Wu X., Shen Z., Wang L. (2024). Contribution Assessment and Accumulation Prediction of Heavy Metals in Wheat Grain in a Smelting-Affected Area Using Machine Learning Methods. Sci. Total Environ..

[177] Huang Y., Zhang N., Ge Z., Lv C., Zhu L., Ding C., Liu C., Peng P., Wu T., Wang Y. (2024). Determining Soil Conservation Strategies: Ecological Risk Thresholds of Arsenic and the Influence of Soil Properties. Eco-Environ. Health.

[178] Jin H., Zhang L., Sun Y., Xu Y., Luo M. (2025). Developing Machine Learning Models for Predicting Cardiovascular Disease Survival Based on Heavy Metal Serum and Urine Levels. Front. Public Health.

[179] Wang F., Wang F., Yang H., Yu J., Ni R. (2023). Ecological Risk Assessment Based on Soil Adsorption Capacity for Heavy Metals in Taihu Basin, China. Environ. Pollut..

[180] Cao Y., Zhao W., Zhong Y., Jiang X., Mei H., Chang Y., Wu D., Dou J., Vasquez E., Shi X. (2024). Effects of Chronic Low-Level Lead (Pb) Exposure on Cognitive Function and Hippocampal Neuronal Ferroptosis: An Integrative Approach Using Bioinformatics Analysis, Machine Learning, and Experimental Validation. Sci. Total Environ..

[181] Lyu T., Tang Y., Cao H., Gao Y., Zhou X., Zhang W., Zhang R., Jiang Y. (2023). Estimating the Geographical Patterns and Health Risks Associated with PM2.5-Bound Heavy Metals to Guide PM2.5 Control Targets in China Based on Machine-Learning Algorithms. Environ. Pollut..

[182] Li K.-Y., Podgorski J., Liang C.-P., Chen J.-S., Chang J.-Y., Berg M. (2026). Groundwater Arsenic in Taiwan: From Mid-20th-Century Crisis to Predictive Models for Risk Mitigation Strategies. Environ. Int..

[183] Akram S., Iltaf B., Shabbir A., Junaid J.A. (2025). Harnessing Multivariate and Machine Learning Tools for Morpho-Physiological Traits to Reveal Maize Genotype Performance against Heavy Metals Stress. J. Soil Sci. Plant Nutr..

[184] Babić Leko M., Mihelčić M., Jurasović J., Nikolac Perković M., Španić E., Sekovanić A., Orct T., Zubčić K., Langer Horvat L., Pleić N. (2022). Heavy Metals and Essential Metals Are Associated with Cerebrospinal Fluid Biomarkers of Alzheimer’s Disease. Int. J. Mol. Sci..

[185] Shi J., Yang Y., Shen Z., Lin Y., Mei N., Luo C., Wang Y., Zhang C., Wang D. (2024). Identifying Heavy Metal Sources and Health Risks in Soil-Vegetable Systems of Fragmented Vegetable Fields Based on Machine Learning, Positive Matrix Factorization Model and Monte Carlo Simulation. J. Hazard. Mater..

[186] Hsieh T.-Y., Chiueh P.-T. (2026). Integrating Multivariate Statistics and AI for Groundwater Heavy Metal Risk Detection: A Case Study from Yilan, Taiwan. Water Pract. Technol..

[187] Pandey U., Madhugiri I., Gadgil C., Gadgil M. (2024). Leveraging Machine Learning to Dissect Role of Combinations of Amino Acids in Modulating the Effect of Zinc on Mammalian Cell Growth. Biotechnol. Prog..

[188] Chaurasia P., Yogarajah P., Ali Mahdi A., McClean S., Kaleem Ahmad M., Jafar T., Kumar Singh S. (2025). Machine Learning and Explainable Artificial Intelligence to Predict and Interpret Lead Toxicity in Pregnant Women and Unborn Baby. Front. Digit. Health.

[189] Lobo G.P., Kalyan B., Gadgil A.J. (2021). Predicting Childhood Lead Exposure at an Aggregated Level Using Machine Learning. Int. J. Hyg. Environ. Health.

[190] Li G.-F., Yu J.-Q., Wang H., Chi H.-F., Lin S.-N., Cai C. (2024). Prediction of heavy metal toxicity and ecological risk based on machine learning methods. Zhongguo Huanjing KexueChina Environ. Sci..

[191] Sakizadeh M., Ahmadpour E., Sharafabadi F.M. (2019). Spatial Analysis of Chromium in Southwestern Part of Iran: Probabilistic Health Risk and Multivariate Global Sensitivity Analysis. Environ. Geochem. Health.

[192] Wen J., Wang C., Liu R., Zhuang R., Liu Y., Li Y., Guo S. (2025). Systemic Inflammation Mediates the Relationship between Urinary Cadmium and Chronic Cough Risk: Findings Based on Multiple Statistical Models. BioMetals.

[193] Li Y., Shi R., Ding S., Guo J., Dai W., Li Y., Li X. (2026). Underestimated Distribution and Health Risks of Arsenic in Soils across the Tibetan Plateau. J. Hazard. Mater..

[194] Luo L., Wang L., Li Y., Cao H., Guo Y., Liao X. (2025). Urban-Rural Inequality in Soil Heavy Metal Health Risks: Insights from Baoding, China. Ecotoxicol. Environ. Saf..

[195] Zhang H., Wan T., Li X., Abbas G., Lu J. (2026). Using Machine Learning to Predict Heavy Metal Concentrations and Induced Health Risks in Drinking Water Distribution Systems from Key Cities in China. Water Res..

[196] Chen X., Zhang C., Yan K., Wei Z., Cheng N. (2023). Risk Assessment of Agricultural Soil Heavy Metal Pollution under the Hybrid Intelligent Evaluation Model. IEEE Access.

[197] Kumar S., Sharma V., Velusamy P. Simulating the Indian Chemical Exposome: A Multi-Modal Transfer Learning Framework for Predicting Heavy Metal Bioaccumulation. Proceedings of the 2026 International Conference on Connected Intelligence for Industrial Applications (CI2A).

[198] Prusty G., Rabi Prasad B., Suman P., Sravani M., Narayana G.V.S. (2025). Biosmart Environmental Technologies Integrating of AI and IoT for Real Time Monitoring of Lead Induced Stress in Pila Globosa. Proceedings of the 2025 International Conference on Next Generation of Green Information and Emerging Technologies (GIET).

[199] Yu K., Fang S., Zhao Y. (2021). Heavy Metal Hg Stress Detection in Tobacco Plant Using Hyperspectral Sensing and Data-Driven Machine Learning Methods. Spectrochim. Acta A Mol. Biomol. Spectrosc..

[200] Hu G., Bakhtavar E., Hewage K., Mohseni M., Sadiq R. (2019). Heavy Metals Risk Assessment in Drinking Water: An Integrated Probabilistic-Fuzzy Approach. J. Environ. Manag..

[201] Sui S., Wang M., Ma W., Wang M., Wang J., Liu K., Jiang F., Guo X., Xing M., Han Q. (2025). Machine Learning Reveals Heavy Metal Migration Pathways in Asia’s Largest Pb-Zn Smelting Region: Soil Pollution Simulation in Jiyuan. Process Saf. Environ. Prot..

[202] Sambanis A., Osiecki K., Cailas M., Quinsey L., Jacobs D.E. (2023). Using Artificial Intelligence to Identify Sources and Pathways of Lead Exposure in Children. J. Public Health Manag. Pract..

[203] Sachchu M.M.H., Hasan M., Hossain M.K., Ahmed S., Eva T.N., Lima M.N.J., Hossain A., Alam M.A. (2025). Heavy Metal Contamination in Pangas Catfish of Farm Ponds Aquaculture in Different Locations in Bangladesh: A Risk Assessment Study. Food Chem. Adv..

[204] Zarate-Cáceres C.R., Iparraguirre-Meza M., Pérez-Venegas C.J., Picoy-Gonzales J.A., Ordoñez-Ccora G., Lacho-Gutiérrez P., Riveros-Laurente K.Y., Diaz-Aranda D.L., Gutiérrez-Iparraguirre G.G., Huarcaya-Taype R. (2026). Integrated Water Quality Assessment and Health Risk Analysis of Heavy Metal and Microbial Contamination in the Ichu River, Peru. F1000Research.

[205] Hussein M.A., Shamma S., Sewilam H.N., Shoeib T., Abdelnaser A. (2025). Spatiotemporal Dynamics and Machine Learning-Based Risk Assessment of Heavy Metal Contamination in Surface Waters and Nile Tilapia in Egypt. Environ. Chall..

[206] Fan L., Chao Y., Yan X., Wang J., Zhuge Y., Lou Y., Wang, Hong P., Yang Q. (2023). Effects of cadmium stress on seed germination characteristics of 58 millet accessions. Pratacultural Sci..

[207] Moon S., Lee J., Yu J.M., Choi H., Choi S., Park J., Choi K., Kim E., Kim H., Kim M.J. (2023). Association between Environmental Cadmium Exposure and Increased Mortality in the U.S. National Health and Nutrition Examination Survey (1999–2018). J. Expo. Sci. Environ. Epidemiol..

[208] Kiełbus M., Wojnicka J., Prystupa A., Nowicki G.J., Urbańczyk A., Prystupa B., Kiciński P., Myśliński W., Ślusarska B., Szmagara A. (2026). Clinical Relevance of Tissue Copper, Selenium, and Cadmium Alterations in Colorectal Cancer. Sci. Rep..

[209] Su F., Zeeshan M., Xiong L.-H., Lv J.-Y., Wu Y., Tang X.-J., Zhou Y., Ou Y.-Q., Huang W.-Z., Feng W.-R. (2022). Co-Exposure to Perfluoroalkyl Acids and Heavy Metals Mixtures Associated with Impaired Kidney Function in Adults: A Community-Based Population Study in China. Sci. Total Environ..

[210] Qiu H., Gui H. (2019). Heavy Metals Contamination in Shallow Groundwater of a Coal-Mining District and a Probabilistic Assessment of Its Human Health Risk. Hum. Ecol. Risk Assess. Int. J..

[211] Nguyen M. (2026). Linear Regression. Regression Techniques for Data Analysis.

[212] Nguyen M. (2026). Generalized Linear Models. Regression Techniques for Data Analysis.

[213] Fahrmeir L., Kneib T., Lang S., Marx B.D. (2021). Structured Additive Regression. Regression: Models, Methods and Applications.

[214] Gillariose J., Joseph J., Chesneau C. (2025). Lasso and Ridge Regression: A Comprehensive Review of Applications and Developments in Machine Learning. Int. J. Data Sci. Anal..

[215] Christensen R. (2025). Logistic Regression, Logit Models, and Logistic Discrimination. Log-Linear Models and Logistic Regression.

[216] Mundlamuri R., Gunnam G.R., Mysari N.K., Pujuri J. (2025). The Evolution of AI: From Classical Machine Learning to Modern Large Language Models. IEEE Access.

[217] Liu J., Gu J., Li H., Carlson K.H. (2020). Machine Learning and Transport Simulations for Groundwater Anomaly Detection. J. Comput. Appl. Math..

[218] Barros R.C., de Carvalho A.C.P.L.F., Freitas A.A. (2015). Decision-Tree Induction. Automatic Design of Decision-Tree Induction Algorithms.

[219] Li H. (2024). Decision Tree. Machine Learning Methods.

[220] Li H. (2024). Boosting. Machine Learning Methods.

[221] Ahlawat S. (2025). Random Forest. Statistical Quantitative Methods in Finance: From Theory to Quantitative Portfolio Management by Samit Ahlawat.

[222] Sun B., Chen H. (2021). A Survey of k Nearest Neighbor Algorithms for Solving the Class Imbalanced Problem. Wirel. Commun. Mob. Comput..

[223] Hastie T., Tibshirani R., Friedman J. (2009). Unsupervised Learning. The Elements of Statistical Learning: Data Mining, Inference, and Prediction.

[224] Hubert M., Debruyne M., Rousseeuw P.J. (2018). Minimum Covariance Determinant and Extensions. WIREs Comput. Stat..

[225] Paalanen P., Kamarainen J.-K., Ilonen J., Kälviäinen H. (2006). Feature Representation and Discrimination Based on Gaussian Mixture Model Probability Densities—Practices and Algorithms. Pattern Recognit..

[226] Li H. (2024). Principal Component Analysis. Machine Learning Methods.

[227] Tilevik A. (2025). Linear Discriminant Analysis. Multivariate Statistics and Machine Learning in R For Beginners.

[228] LeCun Y., Bengio Y., Hinton G. (2015). Deep Learning. Nature.

[229] Ghojogh B., Ghodsi A. (2026). Backpropagation and Optimization in Deep Neural Networks. Elements of Deep Learning.

[230] Ghosh A. (2025). Multilayer Perceptron. Data Science and Cases in Sustainability.

[231] Bishop C.M., Bishop H. (2024). Convolutional Networks. Deep Learning: Foundations and Concepts.

[232] Ghojogh B., Ghodsi A. (2026). Convolutional Neural Networks. Elements of Deep Learning.

[233] Salih A.M., Raisi-Estabragh Z., Galazzo I.B., Radeva P., Petersen S.E., Lekadir K., Menegaz G. (2025). A Perspective on Explainable Artificial Intelligence Methods: SHAP and LIME. Adv. Intell. Syst..

[234] Kamath U., Liu J. (2021). Model Interpretability: Advances in Interpretable Machine Learning. Explainable Artificial Intelligence: An Introduction to Interpretable Machine Learning.

[235] Kursa M.B., Rudnicki W.R. (2010). Feature Selection with the Boruta Package. J. Stat. Softw..

[236] Bobb J.F., Claus Henn B., Valeri L., Coull B.A. (2018). Statistical Software for Analyzing the Health Effects of Multiple Concurrent Exposures via Bayesian Kernel Machine Regression. Environ. Health.

[237] Gennings C., Midya V., Renzetti S., DeFelice N. (2025). Weighted Quantile Sum (WQS) Mixed-Effects model. Methodsx.

[238] Nunkoo R. (2024). Structural Equation Modeling. Encyclopedia of Tourism.

[239] Sun X., Wang H., Guo Z., Lu P., Song F., Liu L., Liu J., Rose N.L., Wang F. (2020). Positive Matrix Factorization on Source Apportionment for Typical Pollutants in Different Environmental Media: A Review. Environ. Sci. Process. Impacts.

[240] Rutherford M.J., Thompson J.R., Lambert P.C. (2012). Projecting Cancer Incidence Using Age-Period-Cohort Models Incorporating Restricted Cubic Splines. Int. J. Biostat..

[241] Bonate P.L. (2001). A Brief Introduction to Monte Carlo Simulation. Clin. Pharmacokinet..

[242] Hunsaker C.T., Graham R.L., Suter G.W., O’Neill R.V., Barnthouse L.W., Gardner R.H. (1990). Assessing Ecological Risk on a Regional Scale. Environ. Manag..

[243] Li D., Zhang C., Pizzol L., Critto A., Zhang H., Lv S., Marcomini A. (2014). Regional Risk Assessment Approaches to Land Planning for Industrial Polluted Areas in China: The Hulunbeier Region Case Study. Environ. Int..

[244] Pizzol L., Critto A., Agostini P., Marcomini A. (2011). Regional Risk Assessment for Contaminated Sites Part 2: Ranking of Potentially Contaminated Sites. Environ. Int..

[245] Hou D., Jia X., Wang L., McGrath S.P., Zhu Y.-G., Hu Q., Zhao F.-J., Bank M.S., O’Connor D., Nriagu J. (2025). Global Soil Pollution by Toxic Metals Threatens Agriculture and Human Health. Science.

[246] Lee H., Jo Y., Jung M., Lee J.H., Kim T.H., Lee J., Kim D.J., Rahmati M., Smith L., Pizzol D. (2026). Heavy Metal Exposure and All Health Outcomes: An Umbrella Review of Meta-Analyses. J. Hazard. Mater..

[247] Alrashdi M.M., Ragazzon-Smith A., Strashnov I., Polya D.A. (2024). Chemical Analysis of Toxic Elements: Total Cadmium, Lead, Mercury, Arsenic and Inorganic Arsenic in Local and Imported Rice Consumed in the Kingdom of Saudi Arabia. Environ. Geochem. Health.

[248] Biswal S.S., Amarnath T., Panigrahi P.K., Biswal N.C., Sharma N., Chakrabarti A., Balas V.E., Martinovic J. (2021). Machine Learning to Diagnose Common Diseases Based on Symptoms. Data Management, Analytics and Innovation: Proceedings of ICDMAI 2020, Volume 2.

[249] Chen X., Liu X., Wu Y., Wang Z., Wang S.H. (2024). Research Related to the Diagnosis of Prostate Cancer Based on Machine Learning Medical Images: A Review. Int. J. Med. Inf..

[250] Manhas J., Gupta R.K., Roy P.P. (2022). A Review on Automated Cancer Detection in Medical Images Using Machine Learning and Deep Learning Based Computational Techniques: Challenges and Opportunities. Arch. Comput. Methods Eng..

[251] Sanmarchi F., Fanconi C., Golinelli D., Gori D., Hernandez-Boussard T., Capodici A. (2023). Predict, Diagnose, and Treat Chronic Kidney Disease with Machine Learning: A Systematic Literature Review. J. Nephrol..

[252] Puntmann V.O. (2009). How-to Guide on Biomarkers: Biomarker Definitions, Validation and Applications with Examples from Cardiovascular Disease. Postgrad. Med. J..

[253] Seriani R., Abessa D.M.S., Pereira C.D.S., Kirschbaum A.A., Abujamara L.D., Buruaem L.M., Félix C., Turatti G.C.R., Prado L.R.G.B., Sousa E.C.P.M. (2014). Blood Parameters of Estuarine and Marine Fish as Non-Destructive Pollution Biomarkers. Pollution and Fish Health in Tropical Ecosystems.

[254] Hanson N., Förlin L., Larsson A. (2009). Evaluation of Long-Term Biomarker Data from Perch (*Perca fluviatilis*) in the Baltic Sea Suggests Increasing Exposure to Environmental Pollutants. Environ. Toxicol. Chem..

[255] Alves M.B., Acioly T.M.d.S., de Sousa A.L., Viana D.C. (2024). Trace metals deposition in otolites of psectrogaster amazonica as environmental indicators in the middle tocantins river. Bol. Conjunt. Boca.

[256] Kadim M.K., Risjani Y. (2022). Biomarker for Monitoring Heavy Metal Pollution in Aquatic Environment: An Overview toward Molecular Perspectives. Emerg. Contam..

[257] Kolawole T.A., Palacios J., Husaini D.C., Nwokocha C.R. (2025). Inflammation and Oxidative Stress Biomarkers in Heavy Metal Toxicity: Bridging the Gap to Personalized Clinical Interventions. J. Appl. Toxicol..

[258] Kiruba B., Narayan P.S.A., Raj B., Raj S.R., Mathew S.G., Lulu S.S., Sundararajan V. (2025). Intervention of Machine Learning in Bladder Cancer Research Using Multi-Omics Datasets: Systematic Review on Biomarker Identification. Discov. Oncol..

[259] Jomova K., Alomar S.Y., Nepovimova E., Kuca K., Valko M. (2025). Heavy Metals: Toxicity and Human Health Effects. Arch. Toxicol..

[260] Ahmad M.F., Ahmad F.A., Zeyaullah M., Bantun F., Babalghith A.O., Ghaith M.M., Zahrani Y., Hummad H., Mozaffar B., Alam M.F. (2026). Heavy Metals in Food: Mechanisms of Toxicity and Migration through Various Routes in Different Foods. Gondwana Res..

[261] Uwizeyimana H., Wang M., Chen W., Khan K. (2017). The Eco-Toxic Effects of Pesticide and Heavy Metal Mixtures towards Earthworms in Soil. Environ. Toxicol. Pharmacol..

[262] Gao F., Shen Y., Brett Sallach J., Li H., Zhang W., Li Y., Liu C. (2022). Predicting Crop Root Concentration Factors of Organic Contaminants with Machine Learning Models. J. Hazard. Mater..

[263] Wu G., Kechavarzi C., Li X., Wu S., Pollard S.J.T., Sui H., Coulon F. (2013). Machine Learning Models for Predicting PAHs Bioavailability in Compost Amended Soils. Chem. Eng. J..

[264] Zhai Y., Zhou L., Qi H., Gao P., Zhang C. (2023). Application of Visible/near-Infrared Spectroscopy and Hyperspectral Imaging with Machine Learning for High-Throughput Plant Heavy Metal Stress Phenotyping: A Review. Plant Phenomics.

[265] Ouyang A., Jiang L., Liu Y., Jiang L., Hao Y., He B. (2015). Determination of Copper and Zinc Pollutants in Ludwigia Prostrata Roxb Using Near-Infrared Reflectance Spectroscopy (NIRS). Appl. Spectrosc..

[266] Khan M.I., Ahmad M.F., Ahmad I., Ashfaq F., Wahab S., Alsayegh A.A., Kumar S., Hakeem K.R. (2022). Arsenic Exposure through Dietary Intake and Associated Health Hazards in the Middle East. Nutrients.

[267] Li C., Xue D., Ren X., Zhao J., Wang H., Zhang S., Premy T., Chen X., Hong M., Zhang C. (2026). Improved ecological risk assessment of heavy metal contamination using machine learning-corrected portable XRF measurements in a high-sulfur mining landscape. Environ. Technol. Innov..

